# Cubic time algorithms of amalgamating gene trees and building evolutionary scenarios

**DOI:** 10.1186/1745-6150-7-48

**Published:** 2012-12-22

**Authors:** Vassily A Lyubetsky, Lev I Rubanov, Leonid Y Rusin, Konstantin Yu Gorbunov

**Affiliations:** 1 Institute for Information Transmission Problems, The Russian Academy of Sciences (Kharkevich Institute), Bolshoy Karetny per. 19, Moscow, 127994, Russia; 2 Faculty of Biology, Moscow State University, Vorob’evy Gory 1/12, Moscow, 119991, Russia

**Keywords:** Phylogenetics, Fast algorithms, Tree inference, Species tree, Tree amalgamation, Tree reconciliation, Supertree, Evolutionary events, Gene duplication, Gene loss, Horizontal gene transfer, Gene gain, Time slices

## Abstract

**Background:**

A long recognized problem is the inference of the supertree *S* that amalgamates a given set {*G*_*j*_} of trees *G*_*j*_, with leaves in each *G*_*j*_ being assigned homologous elements.

We ground on an approach to find the tree *S* by minimizing the total cost of mappings *α*_*j*_ of individual gene trees *G*_*j*_ into *S*. Traditionally, this cost is defined basically as a sum of duplications and gaps in each *α*_*j*_. The classical problem is to minimize the total cost, where *S* runs over the set of all trees that contain an exhaustive non-redundant set of species from all input *G*_*j*_.

**Results:**

We suggest a reformulation of the classical *NP*-hard problem of building a supertree in terms of the global minimization of the same cost functional but only over species trees *S* that consist of clades belonging to a fixed set *P* (e.g., an exhaustive set of clades in all *G*_*j*_). We developed a deterministic solving algorithm with a low degree polynomial (typically cubic) time complexity with respect to the size of input data.

We define an extensive set of elementary evolutionary events and suggest an original definition of mapping *β* of tree *G* into tree *S*. We introduce the cost functional *c*(*G*, *S*, *f* ) and define the mapping *β* as the global minimum of this functional with respect to the variable *f*, in which sense it is a generalization of classical mapping *α*.

We suggest a reformulation of the classical *NP*-hard mapping (reconciliation) problem by introducing time slices into the species tree *S* and present a cubic time solving algorithm to compute the mapping *β*. We introduce two novel definitions of the evolutionary scenario based on mapping *β* or a random process of gene evolution along a species tree.

**Conclusions:**

Developed algorithms are mathematically proved, which justifies the following statements. The supertree building algorithm finds exactly the global minimum of the total cost if only gene duplications and losses are allowed and the given sets of gene trees satisfies a certain condition. The mapping algorithm finds exactly the minimal mapping *β*, the minimal total cost and the evolutionary scenario as a minimum over all possible distributions of elementary evolutionary events along the edges of tree *S*.

The algorithms and their effective software implementations provide useful tools in many biological studies. They facilitate processing of voluminous tree data in acceptable time still largely avoiding heuristics. Performance of the tools is tested with artificial and prokaryotic tree data.

**Reviewers:**

This article was reviewed by Prof. Anthony Almudevar, Prof. Alexander Bolshoy (nominated by Prof. Peter Olofsson), and Prof. Marek Kimmel.

## Background

### Problems in supertree inference

Denote *S* a tree of species or other taxonomic units, proteins, etc. The long recognized problem is to infer a tree *S* that amalgamates a given set {*G*_*j*_} of trees *G*_*j*_, with leaves in each *G*_*j*_ being assigned homologous sequences from an *j*-th family of homologous elements. Only leaf names, not sequences themselves, are considered. Henceforth, assume that leaves in *S* are labeled with species names *x*, leaves in each *G*_*j*_ – with species-gene names *x*-*y* (gene “*y*” exists in species “*x*”); paralogs are allowed. Refer to *S* as a *species tree*, and to each *G*_*j*_ as a *gene tree*.

We elaborate a traditional approach from [1,2] to find the tree *S* such that it minimizes the total cost of mappings of individual gene trees *G*_*j*_ into *S*.

Traditionally, some *cost c*(*GS*) of mapping of a gene tree *G* into a species tree *S* is defined. Choosing a particular definition of *c*(*GS*) (ref. e.g. to [2,3] and see Results) is not essential in terms of solving the classical problem below. For a *given* set {*G*_*j*_} of gene trees the *total cost* is defined as

(1) $$\begin{array}{c} c\left(\left\{G_{j}\right\},S\right)=\sum_{j}c\left(G_{j},S\right)\;\text{or}\\ \;c\left(\left\{G_{j}\right\},S\right)=\sum_{j}k_{j}\cdot c\left(G_{j},S\right) \end{array}$$

where *k*_*j*_ are certain weights. The *classical problem* is to find such *S* that globally minimizes the functional *c*({*G*_*j*_},*S*), where *S* runs over the set of all species trees that contain an exhaustive non-redundant set of species from all input *G*_*j*_. Such *S* is called a *supertree* for the given set {*G*_*j*_}. Denote *V*_0_ a set of all species names occurring in leaves of the input trees *G*_*j*_. Thus, the classical problem is to find the global minimum of cost functional (1) over all species trees *S* that possess the set *V*_0_ of leaf names.

The supertree building problem is *NP*-hard, i.e., any algorithm to solve it must possess an exponential complexity (if *NP* ≠ *P*). Numerous heuristics exist (e.g. in [4-6]), which however do not provide evaluations of the runtime of corresponding algorithms. Unless *NP* = *P*, none of them can possess a polynomial complexity and be proved to find the true global minimum.

We propose a reformulation of the classical problem and develop an effective deterministic algorithm that meets many biological prerequisites (Description of the first algorithm and Results). Namely, the supertree *S* is sought for as a global minimum of (1) but *S* runs over a set of such species trees that mostly contain clades present in input trees *G*_*j*_, [3,7,8]. A set of species names assigned to leaves of a subtree in *G*_*j*_ with the root *v* is called a *clade* (of vertex *v* in *G*_*j*_) and denoted by *cl*(*v*). The set *P* includes all clades from trees *G*_*j*_ and additionally the set of species names *V*_0_. Such *P* is referred to as a *standard* set. Its cardinality has the order of *nm*, where *n* is the number of gene trees, and *m* is the total number of species. For the standard set *P*, the algorithm’s running time is cubic and determined by formula (2) below.

Further, suppose that *cl*(*v*_1_), *cl*(*v*_2_) are the clades of two noncomparable vertices *v*_1_, *v*_2_ in a tree *G*_*j*_, and the intersection *I*(*v*_1_*v*_2_) of these clades is not empty. Optionally, the sets *cl*(*v*_*i*_) – *I*(*v*_1_*v*_2_), (*i*=1, 2) are also included in *P*; and for each vertex *v* that is ancestral to *v*_*i*_ (*i*=1 or *i*=2) but not to another vertex from the pair *v*_1_, *v*_2_, the set *cl*(*v*) – *cl*(*v*_*i*_) is included in *P*. In so doing, *horizontal gene transfers are accounted for* in a species tree, ref. to [9].

If *P* includes any other nonempty subsets of *V*_0_ and its cardinality is arbitrary, the algorithm remains cubic in time but with respect to cardinality |*P*| of set *P*, ref. to formula (3) below.

Therefore, the *non*-*standard problem* formulated in this study consists of finding the global minimum of functional (1) among species trees *S* that have the set of leaves *V*_0_ and a set of clades belonging to a fixed set *P*. Thus, *P* is a parameter of the problem and of the solving algorithm. The algorithm operates equally with any *P*. The solution is also referred to as a *supertree* or a “limited supertree” with respect to *P*.

A mapping of *G*_*j*_ into *S*, as well as defyning any scenario, requires a pre-defined fixed set of elementary *evolutionary events*. The *standard* set (of events) contains only gene duplications and losses. The *extended* set (of events) additionally contains horizontal gene transfers, gains, etc. The list of elementary evolutionary events and their definitions are given in Description of the first algorithm. Henceforth, edges in a species tree are referred to as *tubes* to contrast the difference with the edges in gene trees.

With the standard event set, the algorithm possesses the running time of

(2) $$O\left(n\;m^{3}\left(n+m\right)\right)$$

For simplicity, here we assume that the average number of leaves in given trees *G*_*j*_ is multiple of *m*.

If set *P* has an arbitrary cardinality, the mentioned time has the order of

(3) $$O\left({\left|P\right|}^{3}+{\left|P\right|}^{2}\mathrm{nm}+\left|P\right|m^{3}\right)$$

Let a set {*G*_*j*_} of gene trees and its associated *P be fixed*. A set *V∈P* is defined as *basic* if it is either a singleton set or can be split into two basic sets. Let us introduce the condition

“*V*_0_ is a basic set” (*)

The condition imposes a certain dependency between sets {*G*_*j*_}, *P* and *V*_0_.

With the standard event set and condition (*), the algorithm was mathematically proved [7], which means that it outputs the *true* global constrained *minimum* of the corresponding functional.

It is difficult, however, to mathematically prove the algorithm for the case of the extended event set and/or a relaxation of condition (*). We believe that including horizontal gene transfers still produces valid results [7], and/or condition (*) can be relaxed.

The authors are unaware from published literature of analogous approaches to find a *mathematically proved* supertree in *cubic time*.

In Testing of the algorithms we present testing of the supertree building algorithm with artificial and biological data.

A relevant biological discussion of our approach is provided in [8]. The mapping cost in [3] is similar to the cost from [2] in the case of standard event set.

### Problems in inferring evolutionary scenario

Patterns of gene evolution possess a number of various types of events that co-occur with the species evolution. Identification of these types and assembling elementary events into an evolutionary scenario is crucial for understanding the evolutionary histories of genes, genomes, species, and higher taxonomic lineages, ref. e.g. to [10-12]. Important is to create a model that incorporates all known event types, as well as a model of co-evolution of regulatory systems, genes and species, e.g. [13]. Studies (e.g. in [14]) show the importance of considering suboptimal (in terms of the total cost) solutions in searches, as those might represent optimal scenarios when the costs of elementary events are slightly varied. This problem is partially tackled in Second scenario design: a random process on the graph.

In pioneer papers [2], the *cost c*_0_(*GS*) is defined through the *mapping α*(*G*, *S*) of a given tree *G* into *S* basically as a sum of duplications (pairs <*x*, *z*>, where *α*(*x*)=*α*(*z*)) and gaps (vertices *y*, where there is no *x* for which *y*=*α*(*x*)). For the given *G* and *S*, the mapping *α* can be computed as a global minimum of the functional *c*_0_(*GSf*) that depends on the variable *f* running over a suitable set of mappings *f* of *G* into *S*; such *α* can be obtained in linear time with respect to the size of input data, and *c*_0_(*G,S*) becomes equal to *c*_0_(*G*, *S*, *α*). Definitions of the cost and mapping are closely related to the definition of the *evolutionary scenario*, i.e., a pattern of elementary evolutionary events that a gene undergoes along the branches of tree *S*. An important part of this definition is the choice of allowed elementary evolutionary events and their costs. In [2] the list included only gene duplications and losses. We consider the extended set of elementary evolutionary events listed in the Methods, and the novel definition of cost *c*(*G,S*) (see Computing the total cost of binary gene trees against the species tree).

If horizontal gene transfers are allowed, any mapping algorithm suffers from an intrinsic prohibition of gene transfers across different levels of the species tree. If this prohibition holds, the problem of building the globally minimal (i.e., globally minimizing the cost functional) mapping of *G* into *S* is *NP*-hard.

In order to circumvent the *NP*-hard nature of the problem and develop a polynomial time algorithm to solve it, the concept of time slices in species tree *S* was introduced [3,15,16]. This concept is in some sense related to an earlier approach to date tree vertices [17]. The concept is biologically justified, although its correct definition is still to be developed.

**Figure 1:**
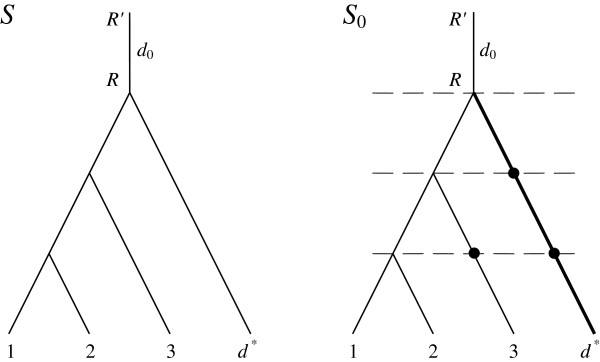
**A transition from tree *S *to tree *S***_**0**_**. **Leaves 1, 2, 3 contain in-group species, leaf d* contains an auxiliary outgroup species. Leaf d* is connected to the root by the *outgroup tube* (shown in bold). All tubes acquire additional vertices during transition to *S*_0_ (right, shown in bold) to delimit time slices (here four slices are separated by dashed lines). Each slice thus contains one segment of the outgroup tube in *S* (left), which forms the *outgroup tubes* in *S*_0_ (each shown in bold). Any such segment, as well as the outgroup leaf-species, are denoted as d*. The root tube *d*_0_ is attached to the root, by analogy with the edge *e*_0_ in a gene tree *G*.

More precisely, edges of *S* can be broken by inserting additional vertices, thus formally producing another tree *S*_0_, Figure 1; in the special case *S*=*S*_0_. It imposes time slices in *S* such that any horizontal transfers are allowed but only within one slice. The algorithm of building time slices [3,15] constructs the tree *S*_0_ such that the *k*-th slice contains all edges distant by the amount of *k* edges from the root. Naturally, any two consecutive edges in *S*_0_ belong to different slices.

Recall that edges in *S*_0_ and *S* are referred to as *tubes* to contrast the difference with the edges in gene trees; inserted vertices split a tube into a succession of new tubes.

The generalization of mapping *α* is proposed for the case of the extended set of elementary evolutionary events listed in Description of the first algorithm. This generalization denotes the mapping *β*. Its definition and details of computing are provided under First scenario design: the event tree. Equivalently, *β* can be defined as the global minimum of the cost *c*(*f*) over a set of mappings *f* of *G* into *S*_0_ (this definition is not provided).

We developed an algorithm that reconciles any gene tree *G* and tree *S*_0_, i.e., computes a rigorous (mathematically proved) minimal mapping *β* of *G* into *S*_0_ in time *O*(|*G*|×|*S*_0_|), which gives *O*(|*S*|^3^) for the time slices building algorithm from [3,15]. Recall that | | is the cardinality of a corresponding set; in terms of trees it is the cardinality of the set of vertices. As above, for simplicity we assume that the average number of leaves in *G* is multiple of *m*. The “mathematically proved” means that *β* is the *true global minimum* of the cost *c*(*f*). The mathematical proof is given in [3,13], and is reproduced with definitions from [3,13] in the later paper [18].

Note that in [16] the biologically important case of the loss of a horizontally transferred gene in the donor (in that study, the case cannot be reduced to a composition of events) is not considered, and the study claims a polynomial runtime of the algorithm yet not specifying the polynomial degree.

### Objectives

One block of objectives is: (i) to formulate the problems and hypotheses in building supertrees and evolutionary scenarios, (ii) to describe the algorithm of solving the non-standard problem of building a supertree (referred to as the *first* algorithm) and to introduce the Super3GL program that implements the algorithm [19] (See Description of the first algorithm and Results), (iii) to compare the program with known supertree inferring tools (Testing of the algorithms).

We describe comparisons with two recognized computer programs in Implementation of the second algorithm, testing against other well-known software tools produced similar results. A rigorous comparison using artificial data implies having a sound algorithm to simulate gene trees on a known species tree topology. This *problem* needs further research and justification, however a particular algorithm was proposed in [8].

The next block objectives is: (iv) to present the concept of the evolutionary scenario based on either mapping *β* or a random process of gene evolution (see First scenario design: the event tree - Stochastic characteristics of the second scenario design), (v) to describe solutions to concomitant problems, viz. computing the originally introduced cost *c*(*GS*) (see Conclusion) and the transition from a polytomous tree to the corresponding binary tree (the “binarization” operation). For convenience, these algorithms in complex are referred to as the *second* algorithm. Implementation of the first algorithm and Implementation of the second algorithm detail the implementations of the first and second algorithms, accordingly [19,20].

## Methods

### Description of the first algorithm

The algorithm is applicable to both an arbitrary set of evolutionary events and an arbitrary set *P*. In this general case the algorithm is heuristic and is tested in Testing of the algorithms (data partially shown). As noted in the Background, the exact condition necessary and sufficient for the algorithm to be mathematically proved is unknown to the authors.

Given is a set of rooted gene trees *G*_*j*_ with allowed polytomies. To pre-process unrooted trees, a simple php script was developed to root trees. The script is available at the Web page [19] and its description is given in Additional file 1.

The first algorithm consists of two phases:

I. building the set {*S*(*V*)| *V*}, where the variable *V* runs over all basic sets (ref. to the Background), and *S*(*V*) is the corresponding (to a given *V*) *basic tree* (this notion is explained below);

II. assembling the set of basic trees *S*(*V*) into the sought-for supertree S*.

Phase I is rigorous (mathematically proved), at least when only gene duplications and losses are considered, and condition (*) is true. However, we operate with the full set of elementary evolutionary events (see Table 1 below), in which case the algorithm is *heuristic*.

**Table 1:** Types of evolutionary events and their costs

***i***	**Condition**	**Name**	**Description**	**Cost**
0	cohered leaf edge *e* and leaf tube *d*	*fin*	evolution of gene *e* ends in species *d*	*c*=0
1	non-cohered leaf edge *e* and leaf tube *d*, *d*≠d*	*tr_fin*	gene *e* evolves into a non-cohered species and transfers without retention to a cohered species	*c*=*с*(tr_without)
2	same as #1 but d=d*	*ga_fin*	gene *e* emerges in a cohered terminal species	*c*=*с*(gain)
3	tube *d* has the single child *d*_1_	*pass*	gene *e* transfers to the next time slice, tube *d*_1_	*c*=*c*(*e*,*d*_1_)
4	edge *e* bifurcates into *e*_1_ and *e*_2_, tube *d* bifurcates into *d*_1_ and *d*_2_	*fork_lr*	*d*≠*d*_0_: gene *e* evolves with speciation into two descendants: *e*_1_ transfers to *d*_1_, *e*_2_ – to *d*_2_; *d=d*_0_: one of the two descendants of gene *e* is absent in the root *R*	*c*=*c*(*e*_1_,*d*_1_)+*c*(*e*_2_,*d*_2_)
5	same as #4	*fork_rl*	*d*≠*d*_0_: gene *e* evolves with speciation into two descendants: *e*_1_ transfers to *d*_2_, *e*_2_ – to *d*_1_; *d=d*_0_: same as #4	*c*=*c*(*e*_2_,*d*_1_)+*c*(*e*_1_,*d*_2_)
6	*d*≠*d*_0_, tube *d* bifurcates into *d*_1_ and *d*_2_	*pass_l*	gene *e* transfers with speciation to *d*_1_ and is lost in *d*_2_	*c*=*c*(*e*,*d*_1_)+*c*(loss)
7	same as #6	*pass_r*	gene *e* transfers with speciation to *d*_2_ and is lost in *d*_1_	*c*=*c*(*e*,*d*_2_)+*c*(loss)
8	*d=d*_0_, tube *d* bifurcates into *d*_1_≠d*, *d*_2_=d*	*nout_l*	gene *e* is present in the root *R*	*c*=*c*(*e*,*d*_1_)
9	*d=d*_0_, tube *d* bifurcates into *d*_1_=d*, *d*_2_≠d*	*nout_r*	same as #8	*c*=*c*(*e*,*d*_2_)
10	*d=d*_0_, tube *d* bifurcates into *d*_1_=d*, *d*_2_≠d*	*out_l*	gene *e* is absent in the root *R*	*c*=*c*(*e*,*d*_1_)
11	*d=d*_0_, tube *d* bifurcates into *d*_1_≠d*, *d*_2_=d*	*out_r*	same as #10	*c*=*c*(*e*,*d*_2_)
12	edge *e* bifurcates into *e*_1_ and *e*_2_, *d*≠d* and genes *e*_1_ and *e*_2_ do not undergo the events *out_l* or *out_r*	*dupl*	gene *e* in *d* duplicated	*c*=*c*(*e*_1_,*d*)+*c*(*e*_2_,*d*)+*c*(dupl)
13	same as #12 but *d=d*_0_ and at least one of the genes *e*_1_ or *e*_2_ undergoes the events *out_l* or *out_r*	*dup*0	one of the descendants of *e* is absent in the root *R*	*c*=*c*(*e*_1_,*d*)+*c*(*e*_2_,*d*)
14	edge *e* bifurcates into *e*_1_ and *e*_2_, d=d*	*outd*	gene *e* is duplicated in the outgroup	*c*=*c*(*e*_1_,*d*)+*c*(*e*_2_,*d*)
15	edge *e* bifurcates into *e*_1_ and *e*_2_, *d*≠d*, *d*≠*d*_0_	*tr*1	one copy *e*_1_ of *e* from *d* transfers to *d' ~ d*, *d'* ≠ d*, another copy *e*_2_ of *e* retains in *d*	*с*=*c*(*e*_1_,*d'*)+*c*(*e*_2_,*d*)+*c*(tr_with) (minimization over *d'*, if uncertainty select one closest to *d*)
16	same as #15	*tr*2	one copy *e*_2_ of *e* from *d* transfers to *d' ~ d*, *d'* ≠ d*, another copy *e*_1_ of *e* retains in *d*	*с*=*c*(*e*_2_,*d'*)+*c*(*e*_1_,*d*)+*c*(tr_with) (minimization over *d'*, if uncertainty select one closest to *d*)
17	edge *e* bifurcates into *e*_1_ and *e*_2_, d=d*	*ga*1	gene *e*_1_ emerges in the species *d' ~ d*	*с*=*c*(*e*_1_,*d'*)+*c*(*e*_2_,*d*)+*c*(gain) (minimization over *d'*)
18	same as #17	*ga*2	gene *e*_2_ emerges in the species *d' ~ d*	*с*=*c*(*e*_2_,*d'*)+*c*(*e*_1_,*d*)+*c*(gain) (minimization over *d'*)
19	*e*≠*e*_0_*, d*≠d*, *d*≠*d*_0_, *d* is not terminal	*sl*	gene *e* stops functioning	*c*=*c*(*e*,d*)+*c*(sleep)
20	*e=e*_0_, d=d*	*ga_big*	gene *e*_0_ emerges in *d' ~ d* as a common ancestor of all *G*_*i*_	*с*=*c*(*e*_0_,*d'*)+*c*(gain_big) (minimization over *d'*)
21	*d*≠d*, *d*≠*d*_0_	*tr_pass*	gene *e* transfers without retention to *d' ~ d, d'* ≠ d*, that produces the single descendant *d'*_1_, and then transfers to *d'*_1_	*c*=*c*(*e*,*d'*_1_)+*c*(tr_without) (minimization over *d'*, if uncertainty select one closest to *d*)
22	*e*≠*e*_0_*, d*=d*	*ga_pass*	gene *e* emerges in *d' ~ d* that produces the single descendant *d'*_1_, and then transfers to *d'*_1_	*c*=*c*(*e*,*d'*_1_)+*c*(gain) (minimization over *d'*)
23	edge *e* bifurcates into *e*_1_ and *e*_2_, *d*≠d*, *d*≠*d*_0_	*tr*_*lr*	gene *e* transfers without retention to *d' ~ d, d'* ≠ d*, that bifurcates into *d'*_1_ and *d'*_2_, then *e*_1_ transfers to *d'*_1_, and *e*_2_ – to *d'*_2_	*с*=*c*(*e*_1_,*d'*_1_)+*c*(*e*_2_,*d'*_2_)+ *c*(tr_without) (minimization over *d'*, if uncertainty select one closest to *d*)
24	same as #23	*tr*_*rl*	gene *e* transfers without retention to *d' ~ d, d'* ≠ d*, that bifurcates into *d'*_1_ and *d'*_2_, then *e*_1_ transfers to *d'*_2_, and *e*_2_ – to *d'*_1_	*с*=*c*(*e*_1_,*d'*_2_)+*c*(*e*_2_,*d'*_1_)+ *c*(tr_without) (minimization over *d'*, if uncertainty select one closest to *d*)
25	*e*≠*e*_0_, edge *e* bifurcates into *e*_1_ and *e*_2_, *d*≠d*	*ga*_*lr*	gene *e* emerges in species *d' ~ d* that bifurcates into *d'*_1_ and *d'*_2_, then *e*_1_ transfers to *d'*_1_, and *e*_2_ – to *d'*_2_	*с*=*c*(*e*_1_,*d'*_1_)+*c*(*e*_2_,*d'*_2_)+*c*(gain) (minimization over *d'*)
26	same as #25	*ga*_*rl*	gene *e* emerges in species *d' ~ d* that bifurcates into *d'*_1_ and *d'*_2_, then *e*_1_ transfers to *d'*_2_, and *e*_2_ – to *d'*_1_	*с*=*c*(*e*_1_,*d'*_2_)+*c*(*e*_2_,*d'*_1_)+*c*(gain) (minimization over *d'*)
27	*d*≠d*, *d*≠*d*_0_	*tr*_*l*	gene *e* transfers without retention to species *d' ~ d, d'* ≠ d* that bifurcates into *d'*_1_ and *d'*_2_, and then transfers to *d'*_1_ and is lost in *d'*_2_	*с*=*c*(*e*,*d'*_1_)+*c*(tr_without)+ *c*(loss) (minimization over *d'*, if uncertainty select one closest to *d*)
28	same as #27	*tr*_*r*	gene *e* transfers without retention to species *d' ~ d, d'* ≠ d* that bifurcates into *d'*_1_ and *d'*_2_, and then transfers to *d'*_2_ and is lost in *d'*_1_	*с*=*c*(*e*,*d'*_2_)+*c*(tr_without)+ *c*(loss) (minimization over *d'*, if uncertainty select one closest to *d*)
29	*e*≠*e*_0_*, d*=d*	*ga_l*	gene *e* emerges in species *d' ~ d* that bifurcates into *d'*_1_ and *d'*_2_, and then transfers to *d'*_1_ and is lost in *d'*_2_	*с*_1_=*c*(*e*,*d'*_1_)+*c*(gain)+*c*(loss) (minimization over *d'*)
30	same as #29	*ga_r*	gene *e* emerges in species *d' ~ d* that bifurcates into *d'*_1_ and *d'*_2_, and then transfers to *d'*_2_ and is lost in *d'*_1_	*с*_1_=*c*(*e*,*d'*_2_)+*c*(gain)+*c*(loss) (minimization over *d'*)
31	edge *e* bifurcates into *e*_1_ and *e*_2_, *d*≠d*, *d*≠*d*_0_	*tr_dupl*	gene *e* transfers without retention to species *d' ~ d, d'* ≠ d*, and then duplicates	*c*=*c*(*e*_1_,*d'*)+*c*(*e*_2_,*d'*)+ *c*(tr_without)+*c*(dupl) (minimization over *d'*, if uncertainty select one closest to *d*)
32	edge *e* bifurcates into *e*_1_ and *e*_2_, *e*≠*e*_0_*, d*=d*	*ga_dupl*	gene *e* emerges in species *d' ~ d,* and then duplicates	*c*=*c*(*e*_1_,*d'*)+*c*(*e*_2_,*d'*)+*c*(gain)+ *c*(dupl) (minimization over *d'*)
33	edge *e* bifurcates into *e*_1_ and *e*_2_, *d*≠d*, *d*≠*d*_0_	*tr*_*double*	gene *e* transfers without retention to species *d' ~ d, d'*≠d*, then its copy *e*_2_ transfers to *d” ~ d, d”* ≠ *d”*, *d”* ≠ d*, and copy *e*_1_ – to *d'*; or vice versa replacing *d'* with *d"* and *e*_1_ with *e*_2_	*c*=*c*(*e*_1_,*d'*)+*c*(*e*_2_,*d"*)+ *c*(tr_without)+*c*(tr_with) (minimization over pair <*d', d"* >, if uncertainty select a pair closest to *d* as per the sum of distances)
34	*e*≠*e*_0_, edge *e* bifurcates into *e*_1_ and *e*_2_, d=d*	*ga*_*double*	gene *e* emerges in species *d' ~ d,* then its copy *e*_2_ transfers to *d" ~ d, d"* ≠ *d'*, and copy *e*_1_ retains in *d'*; or vice versa replacing *d'* with *d"* and *e*_1_ with *e*_2_	*c*=*c*(*e*_1_,*d'*)+*c*(*e*_2_,*d"*)+*c*(gain)+ *c*(tr_with) (minimization over pair <*d’, d"* >)

Consider *i* as the number of the event (and the row number) in a fixed enumeration pattern; “Condition” defines the applicability of the event to current pair <*e*, *d* >; “Name” is the event type; “Description” is the event synopsis; “Cost” contains formulas to compute the costs of scenarios initiated from an event in a current row. A notation *d* ~ *d'* designates that “tubes *d* and *d'* differ and belong to the same time slice”. The constants *c*(dupl), *c*(loss), *c*(gain), *c*(gain_big), *c*(tr_without), *c*(tr_with), *c*(sleep) define the costs of individual events and constitute parameters of the algorithm.

If *V*_0_ is a basic set then *S*(*V*_0_) can already be considered an outcome of the algorithm (omitting Phase II). However, our experiments show that engaging Phase II improves the result quality.

I) The first phase (Phase I) consists of five steps:

I.1) *Optional tree pruning*. An inextensible subtree of *G*_*j*_ with all leaves belonging to a species *s* is called the *occurrence* of species *s* (in *G*_*j*_). For each species *s* that occurs less than *p* times (a parameter of the algorithm) in the given set {*G*_*j*_} of gene trees, every gene of this species is removed from all *G*_*j*_. After such trimming in each *G*_*j*_ “pendant” edges are removed together with their origins. Next, all gene trees that become empty or contain only one species are also removed. Such a reduced set of gene trees is still denoted by {*G*_*j*_}. This step is trivial but might be useful.

I.2) *Composing the set P of species sets.* The set *P* (refer to its definition in the Background) is built and ordered by ascending the cardinality of constituent sets.

**Figure 2:**
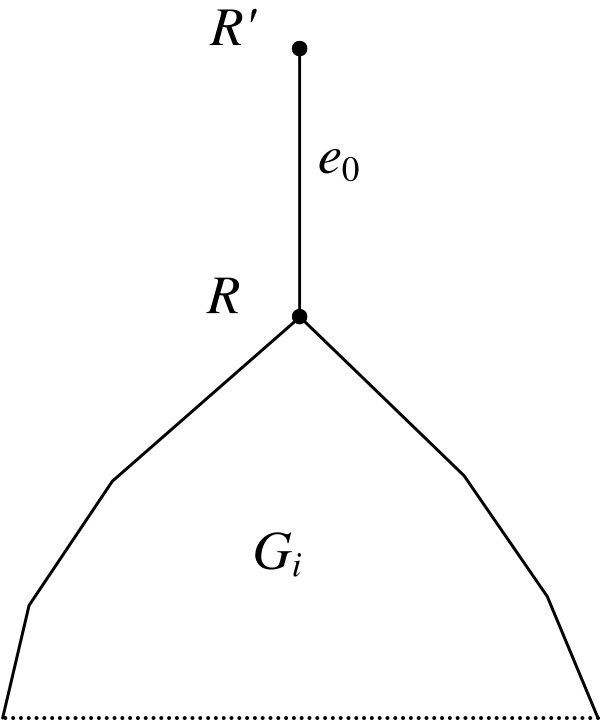
**An additional “root” edge between the “super-root” *R' *and the initial root *R *in tree *G***_***j***_**. **This root is used to define the set *R*(*V*), since the vertex *R'* can be good. The root edge *e*_0_ is analogous to the root tube *d*_0_ in Figure 1.

I.3) *Constructing the set of “good” vertices.* Let *G*_*j*_ be binary. Given a set *V***∈***P*, a non-root vertex *v* of tree *G*_*j*_ is called *good* (with respect to *V*) if

(4) $$\mathrm{cl}\left(v\right)\subseteq V,\;\mathrm{cl}\left(v'\right)⊈V$$

where *v'* is the parent vertex of *v*. The root of the tree is considered good if the first condition in (4) is true. Now let *G*_*j*_ be polytomous. We assume that *G*_*j*_ contains an additional edge *e*_0_ located upwards from the root as shown in Figure 2. Given a set *V***∈***P*, a vertex *v* of tree *G*_*j*_ is called *good* (with respect to *V*) if at least one of its children obeys condition (4) or the first condition in (4) if *v* is the super-root. For each set *V***∈***P*, the *set R*(*V*) of good vertices in all source gene trees *G*_*j*_ is composed. If a binary tree *G*_*j*_ is also considered polytomous, these two definitions give, generally defining, different sets of good vertices but of equal cardinality. It is enough, as only the cardinality of the set *R*(*V*) is considered further.

I.4) *Finding basic sets and their partitions in the set P.* For each fixed non-singleton basic set *V* (in *P*), all partitioning variants are considered, i.e., all variants defined by the equality *V = V*_1_+*V*_2_, where non-empty disjoint sets *V*_1_, *V*_2_ are themselves basic.

**Figure 3:**
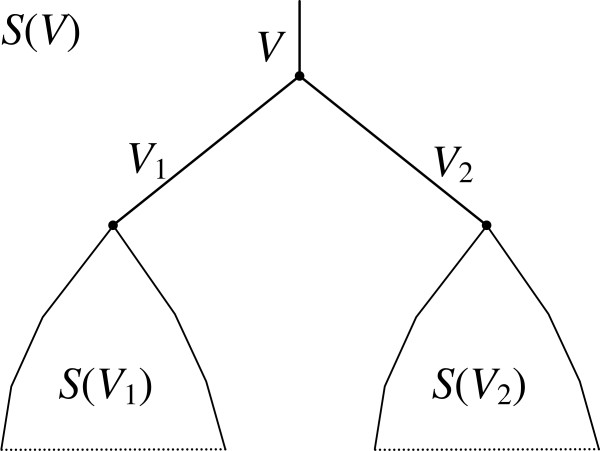
**Tree *S*(*V*) for a fixed partition *V *= *V***_**1**_**+ *V***_**2**_**. **Here *V*, *V*_1_, *V*_2_ designates both the corresponding vertex and the edge upwards from this vertex. Trees *S*(*V*_1_) and *S*(*V*_2_), as well as their costs *c*(*V*_1_) and *c*(*V*_2_), are already known from induction, and *C*_*d*_ + *C*_*l*_ corresponds to evolutionary events in edges of *V*, *V*_1_, *V*_2_. Those are not known from induction and should be computed separately, as defined below in the text.

I.5) *Building basic trees S*(*V*) *and computing their costs.* For each basic set *V*, the basic tree *S*(*V*) along with its cost *c*(*V*) is defined and computed by induction. The tree *S*(*V*) for a singleton set *V* consists of one root-leaf vertex assigned a species from *V*; the cost *c*(*V*) of this *S*(*V*) is zero. The induction step for a fixed *V*: for each partition variant *V = V*_1_+*V*_2_ the value *c*(*V*_1_,*V*_2_) = *c*(*V*_1_) + *c*(*V*_2_) + *C*_*d*_ + *C*_*l*_ is computed, and the minimum (over all partitions of *V*).

$$c\left(V\right)=\min\left\{c\left(V_{1},V_{2}\right)|V=V_{1}+V_{2}\right\}$$

is found, where *C*_*d*_ is the total cost of duplications on edge *V* (we equally denote by *V* the set of leaf species, the root edge and the root vertex of the corresponding subtree), and *C*_*l*_ is the total cost of losses on edges *V*_1_ and *V*_2_, see Figure 3. Both *C*_*d*_ and *C*_*l*_ are defined below.

A partition <*V*_1_,*V*_2_> that minimizes the functional *c*(*V*) over all partitions of *V* is called the *minimal partition* (of *V*). Once the minimal partition is found, the tree *S*(*V*) is obtained by joining trees *S*(*V*_1_) and *S*(*V*_2_) and rooting them at the joint vertex and edge, as shown in Figure 3.

Thus, to calculate the total costs *C*_*d*_ and *C*_*l*_, a *set r*(*V*_1_,*V*_2_) of vertices *v* in all *G*_*j*_ is constructed such that one child vertex of *v* belongs to *R*(*V*_1_), and the other – to *R*(*V*_2_) (if *v* is binary). A polytomous vertex *v* is included in *r*(*V*_1_,*V*_2_) if *v* possesses at least one child satisfying (4) for *V*_1_ and one satisfying (4) for *V*_2_. The total cost of duplications on edge *V* is calculated as *C*_*d*_ = *c*_*d*_ · (|*R*(*V*_1_)|+|*R*(*V*_2_)|–|*R*(*V*)|–|*r*(*V*_1_,*V*_2_)|), where | | denotes the cardinality of a set, and *c*_*d*_ is the cost of one duplication (the algorithm parameter). The total cost of losses in edges *V*_1_ and *V*_2_ is calculated as *C*_*l*_ = *c*_*l*_ · (|*R*(*V*_1_)|+|*R*(*V*_2_)|–2|*r*(*V*_1_,*V*_2_)|), where *c*_*l*_ is the cost of one loss (the algorithm parameter).

Additionally, the *weight* of the tree *S*(*V*) is calculated with the formula

$$w\left(V\right)=\left(1+\frac{\mathrm{ca}}{m}\right)\frac{\left(c\left(V^{'}\right)-c\left(V\right)\right)}{c\left(V^{'}\right)}$$

where *a* is the number of leaves in *S*(*V*), *m* is the total number of species, and *c* is the algorithm parameter (default is *c*=10). Here the partition *V'* is closest to the minimal partition in terms of the cost; if no other partition exists, it is assumed that *w*(*V*) = 1. The weights are used at Phase II of the algorithm.

Phase I (steps I.1-I.5) ends with removing all basic trees containing less than 3 leaves. The obtained set of weighted basic trees is the outcome of Phase I of the first algorithm.

Operating time of Phase I for the standard *P* has the order of *O*((*n·m*)^3^), and for any *P* – the order is *O*(|*P*|^3^ + |*P*|^2^*nm*). A rigorous cubic complexity and mathematical proof (in the special common case) are the advantages of the Phase I algorithm comparing to known heuristic methods.

II) The second phase of the first algorithm (Phase II). This phase is heuristic. For any species tree *S* with the leaves-species set *W* and the basic tree *S*(*V*), define the *cost c*(*S*(*V*),*S*) as the cost of mapping *α* or *β* of the tree *S'*(*V*) into *S* (the cost of *β* is defined in see Computing the total cost of binary gene trees against the species tree below). Here, *S'*(*V*) is obtained from *S*(*V*) by pruning all leaves containing species outside *W* together with their edges.

The *cost c*(*S*) of any species tree *S* is defined as the sum of costs *c*(*S*(*V*),*S*) over all basic trees *S*(*V*), or, optionally, each cost is multiplied by *w*(*V*). Thus,

$$c\left(S\right)=\sum_{S\left(V\right)}w\left(V\right)\cdot c\left(S\left(V\right),S\right)$$

where summation is done over all basic trees *S*(*V*) or, equally, over all basic sets *V* with cardinality higher than or equal to 3. As above, let *V*_0_ be the set of all species contained in leaves of all *G*_*j*_.

*The initial step of Phase II.* The algorithm enumerates all triplet-leaved trees *S* with three leaves-species from *V*_0_ and selects one with the minimal cost *c*(*S*). This *S* constitutes the seed partial supertree in the below procedure.

**Figure 4:**
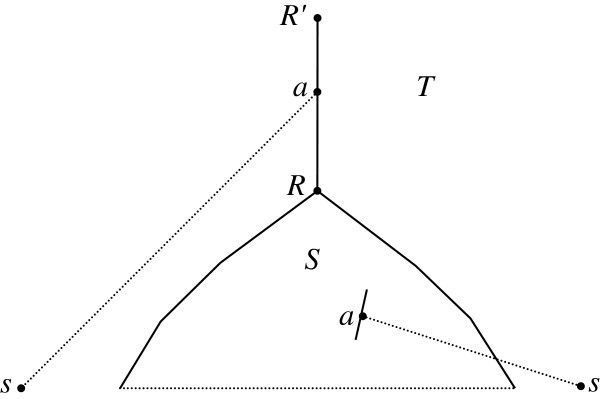
**Two possibilities of inserting a new vertex *a *connected by an edge with a new species *s.***

*The inductive step of Phase II.* In the current partial supertree *S* with the set *W* of leaves-species (a subset of *V*_0_), each edge is attempted for insertion of a new vertex *a* connected to a species *s* from *V*_0_, and for placing a new root *a* above the current root, Figure 4. Among such possible extensions *T* of *S*, we choose the tree *T* with the minimal cost *с*(*T*); it supersedes the current partial supertree *S*. Extensions are attempted until all species from *V*_0_ are added to the current tree *S*, and the algorithm halts. The eventual *S* is the sought-for supertree S*. The end of Phase II.

Additional file 1 contains a more detailed description of Phase II, including the assessment of *vertex reliability* in the final supertree and overall supertree *reliability*. It also presents a simplified version of Phase II.

### The second algorithm: reconciliation of gene and species trees and building evolutionary scenarios

Given are a set {*G*_*j*_} of rooted polytomous gene trees (paralogs are allowed), a rooted binary species tree *S* and a tree *S*_0_ obtained from *S* by inserting one or several vertices into tubes to delimit time slices, Figure 1. Each *G*_*j*_ and the tree *S*_0_ are attributed the root edge *e*_0_ and the root tube *d*_0_ as depicted in Figure 2. If the index *j* is irrelevant, *G*_*j*_ is equivalent to *G*.

The set of species is fixed, with one “accessory” outgroup leaf d* added to the tree root. For the species tree, the notations of terminal tubes, leaves and species *x* (including the outgroup) are unified to define identical objects. For gene trees, the identical objects are terminal edges, leaves and leaf notations *x*-*y*. The correspondence between leaf notations *x*-*y* in *G* and *x* in *S* and *S*_0_ is fixed as the gene-species correspondence (gene “*y*” exists in species “*x*”).

The second algorithm refers to a complex of algorithms to solve four separate problems as described below in Computing the total cost of binary gene trees against the species tree - Stochastic characteristics of the second scenario design.

#### Ordering used in the algorithm

All gene trees *G*_*j*_ are enumerated (index *j* can be omitted). Under a fixed *j*, triplets <*e*,*d*,*i* > are enumerated as follows. Edges *e* in *G* are visited in the order of descending distance from the root (from deeper to shallower levels), and from left to right within the same level. Tubes *d* in *S*_0_ are visited in the order of descending the level of the time slice (upwards to the root). Within a slice, for each *e ≠ e*_0_ tubes are visited from left to right starting from the outgroup d*, and for the root edge *e*_0_ the left-to-right visiting ends with the outgroup d*. Here d* is a segment of the outgroup tube in tree *S* that falls within the current time slice. Next, the 35 types of gene evolution events *i* are visited in the order specified in Table 1, with *i* running from 0 to 34.

All trees *G* considered (see Conclusion) are binary. Encountered polytomous trees are binarized. The binarization procedure is described in Additional file 2.

#### Computing the total cost of binary gene trees against the species tree

In Table 1, each row provides the event name and description (third and fourth columns, respectively), and the last column defines the cost of <*e*,*d*,*i*>.

**Figure 5:**
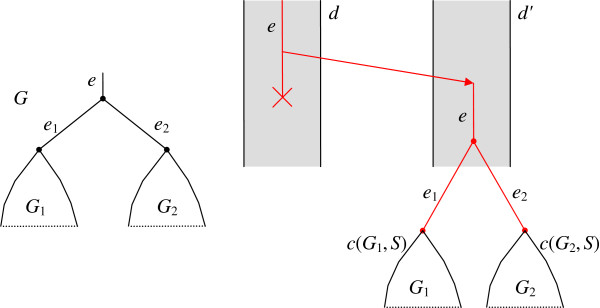
**The inductive step in computing the cost *c*(*G*,*S*). **On the left is an illustration of assembling the tree *G* from subtrees *G*_1_ and *G*_2_. Here *е*_1_ is the root edge in *G*_1_, *е*_2_ – the root edge in *G*_2_ (the root edges belong to their corresponding trees), and *е* – the root edge in *G*. Figure on the right illustrates an embedding of *G* into *S*. Costs *c*(*G*_1_,*S*) and *c*(*G*_2_,*S*) are computed with induction. Then *c*(*G*,*S*) = *c*(*G*_1_,*S*) + *c*(*G*_2_,*S*) + *x*, here *х=с*(loss)+*c*(transfer)+*c*(dupl), and summands are parameters of the algorithm (elementary event costs).

Let *j* specify the tree *G*, *e* run over its edges, and *d* run over tubes in *S*_0_. Define

(5) $$c_{\min}\left(e,d,j\right)=c_{\min}\left(e,d\right)=\min_{i}c\left(e,d,i\right)$$

where *с*(*e*,*d*,*i*) is the cost specified in the last column of Table 1 if the current pair <*e*,*d>* satisfies the condition defining the particular event type. In computing *с*_min_(*e*,*d*) the arguments <*e*,*d>* are enumerated according to the ordering specified in The second algorithm: reconciliation of gene and species trees and building evolutionary scenarios. Figure 5 exemplifies an induction step.

The minimum of (5) is attained at the “*minimal*” event (the “*minimal*” row in Table 1) *i*. Certain events imply the minimization over the variable tube *d'* or the pair of tubes <*d'*,*d">*; the minimal value of the variable is referred to as the “*minimal parameter*”.

The value *с*_min_(*e*_0_, *d*_0_) is *denoted* as *c*(*G*, *S*), recall that *G* = *G*_*j*_. The value $\sum_{j}c_{\min}\left(e_{0},d_{0},j\right)$ is denoted as *c*({*G*_*j*_}, *S*) and referred to as the *total cost* of the input set {*G*_*j*_} of binary gene trees against the tree *S*. The *total cost* of the supertree S* is denoted as *c*({*G*_*j*_}, *S* *).

The value *с*_min_(*e*, *d*) can be interpreted as a “conditional cost”, i.e. the cost of an optimal evolutionary scenario if it initiates from edge *e* in tube *d* and evolves into terminal leaves with cohered pairs of genes-species.

#### First scenario design: the event tree

Each tree *G* (or its binarization *G'*) is associated with the *first scenario* (the event tree) *T* of the evolution of gene *G* along the species tree *S*_0_. The tree vertices correspond to certain pairs <*e*,*d*>, the root – to the pair <*e*_0_,*d*_0_>, the leaves – to pairs formed with a terminal edge and a terminal tube obeying the “species-gene” relation. The tree edges can be unary (ordinary) or binary, i.e., pairs of unary edges originated from a single vertex. The algorithm of constructing *T* over *G* is similar to the binarization procedure detailed in Additional file 2.

During the forward run (described in Computing the total cost of binary gene trees against the species tree) each pair <*e*,*d*> is assigned the minimal event *i* according to (5) and its minimal parameters. The backward run starts from the pair <*e*_0_,*d*_0_>. At each step either a binary edge is projected from vertex <*e*,*d*> into vertices denoted as <*е*_1_,*d'*_1_> and <*е*_2_,*d'*_2_> (case 1), or a unary edge is projected into vertex <*e*,*d'*> (case 2), where *d'*_1_, *d'*_2_, *d'* are the minimal parameters. The edge is tagged with the event name *i*. Case 1 implies a bifurcation resulted from the minimal event.

By definition, the cost of the first scenario *T* is the cost of the input tree *G* against *S*_0_, i.e. *c*(*T*) = *c*(*G*, *S*_0_). It can be detailed with the amounts of different event types inferred in tubes of the species tree, the total amount of events, the individual event costs, etc.

**Figure 6:**
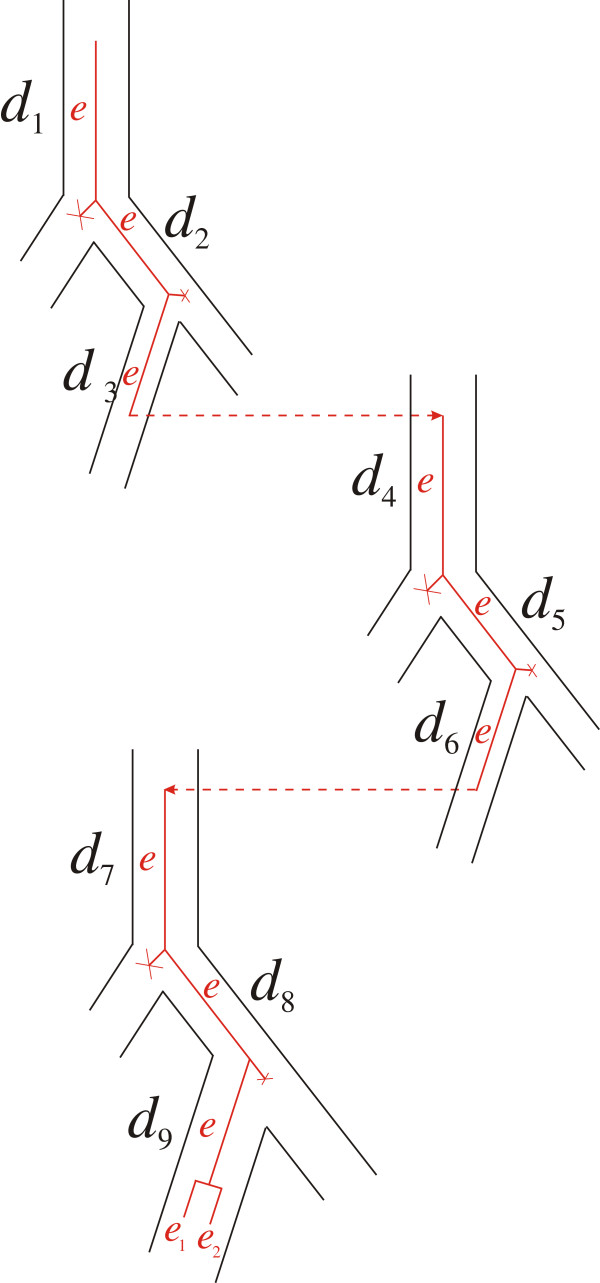
**An example of *β*(*e*) value. **Edge *e* may cross different time slices (not shown), undergoes several speciation events with a loss, two horizontal transfers without retention, and, importantly, terminates with a duplication event.

The *mapping β* is equivalent to *T* , and the cost of *β* is equal to the cost of *T* as substantiated below. It is easy to show that for each *е* in *G* there are vertices in *T* of the form <*e*, *d* > with different tubes *d*. Each such tube *d*_1_,…, *d*_*l*_ is associated in *T* with the *unique* corresponding event *i*_*t*_ that occurred on edge *e* inside tube *d*_*t*_ (such *i*_*t*_ tags the unique edge originated from vertex <*e*, *d*_*t*_ > in *T*). By definition, *β*(*e*)={*d*_1_, …, *d*_*t*_,…, *d*_*l*_}. The set *β*(*e*) can be interpreted as a path. Consider first *d*_1_ that is closest to the root in *S*_0_. If tubes *d*_*t*_ and *d*_*t*+1_ are comparable then *d*_*t*_ is closer to the root, otherwise *d*_*t*+1_ accepts a transfer from *d*_*t*_ (Figure 6) or *d*_*t*+1_ is a child of the accepting tube. The set *β*(*e*) forms in *S*_0_ a connected path defined by the scenario *T* and consisting of repetitions of edge *e* and transfers without retention. This definition of *β*(*e*) requires a clarification: events *i*_*t*_ are determined by *β*(*e*) and *S*_0_, except for the last event *i*_*l*_. Therefore, *β*(*e*) can be expressed as *β*(*e*)={*d*_1_, …, *d*_*l*_; *i*_*l*_}. For mapping *β* let us define *c*(*β*) = *c*(*T*).

The event tree *T* can be easily recovered with a known *β*, which is however not of interest because *T* is used as the first scenario. Note that *β* is the global minimum of the cost functional *c*(*f*), where *f* is any admissible distribution of edges in *G* along tubes in *S*_0_; we omit exact definitions here.

#### Second scenario design: a random process on the graph

In First scenario design: the event tree, the scenario *T* is constructed during consecutive selection of minimal events in the tubes. However, the discarded alternatives may represent events with just slightly higher costs. As true event costs are unknown, it becomes an important consideration. We describe a novel approach to construct the scenario as a random process on the graph, which allows us to take suboptimal scenarios into account.

Fix a natural number *k* (the “degree of ramification”, the algorithm parameter).

For each *G*, construct a *directed acyclic graph* (DAG) *R* with unary and binary edges, vertices corresponding to pairs <*e*, *d* >, and the root <*e*_0_, *d*_0_ >. The edges are tagged with event names *i*_1_, …, *i*_*l*_ (where *l* ≤ *k*) from Table 1. During the forward run of the algorithm, unlike with the first scenario (event tree) *T*, not one but *k* “best” (in terms of the cost) unary or binary edges are projected from each vertex <*e*, *d* > and tagged with the event *i*, i.e. *i* takes *k* or less values at each vertex. Each edge is assigned conditional probability *p*_*i*_ and unconditional probability *p*(*e*, *d*, *i*) of undergoing evolutionary events *i.* Under *k* = 1 the evolution is deterministic, i.e., the probabilities are either 0 or 1, and edges receiving the probability of 1 constitute the first scenario *T.*

Leaf pairs <*e*, *d* > cohered by the “species-gene” correspondence constitute the leaves in DAG, with no outgoing edges. A non-cohered pair <*e*, *d* > projects an edge into the cohered pair <*e*, *х* >, where *x* is the tube that terminates with the species assigned to the leaf *e*. This edge is tagged with the probability *p*_*i*_=1 and the row number 1 (Table 1). A pair <*e*, d*> also projects an edge into a cohered pair <*e*, *х* >; the edge is tagged with *p*_*i*_=1 and the row number 2 (Table 1).

The above paragraph describes the start of induction in the construction of DAG. The induction step is more sophisticated and is described in Additional file 2.

Intuitively, DAG describes the evolutionary branching of a gene described by the tree *G* along a species described by the tree *S*. For each *G*, the value *p*(*e*, *d*, *i*) assigned to the DAG edge <*e*, *d*, *i* > is a probability of inclusion of the edge into the event tree. Starting from vertex <*e*_0_, *d*_0_ > and arriving into <*e*, *d* >, choose its *i*-th outgoing edge with the probability *p*_*i*_. If a unary edge is chosen, proceed to its terminus; if a binary edge is chosen, the process bifurcates into the termini of the edge.

Note that the lower the cost *c*(*e*, *d*, *i*), the higher the probability *p*_*i*_. For the second scenario, the algorithm computes not the cost but the expectation of the number and total cost of various event types. The expectations depend on parameter *k*, which default value is 10. Computer simulations show that higher *k* produce similar expectations.

The first scenario is the best in terms of the cost, the second scenario incorporates a number of good solutions (the threshold set indirectly by *k*). Under *k* = 1 the scenarios coincide, and cost expectations coincide with the costs. Under *k* > 1 the second scenario is a refinement of the first scenario. E.g., if duplications in the root tube are absent in the best scenario but present in suboptimal solutions, the second scenario will show their expectation already at *k* = 10.

#### Stochastic characteristics of the second scenario design

Denote an *I-type* a fixed set *I* of tags selected by the user. The third column of Table 2 contains the following tags: gene gain (*gain*), origin of the common ancestor of all genes (*gain_big*), gene duplication (*dupl*), gene loss (*loss*), gene transfer from a tube with retention (*tr*^*+*^*o*), gene transfer from a tube without retention (*tr*^*–*^*o*), gene transfer into a tube with retention (*tr*^*+*^*i*), gene transfer into a tube without retention (*tr*^*–*^*i*), loss of the transferred copy in the donor (*loss*^*–*^). Other tags can be added to define event types in terms of DAG.

**Table 2:** Definitions of events in the second scenario design (in the DAG)

***i***	**Termini of the edge projected from <*e*, *d*>**	**Triplets**
0	Edge is not projected (induction ends)	None
1	<*e*,*х*> (*х* is a leaf cohered with *e*; induction ends)	<*tr*^*–*^*o,e,d*>, <*tr*^*–*^*i,e,х*>, <*loss*^*–*^*,e,d*>
2	same as #1	<*gain,e,х*>
3	<*e,d*_1_>	None
4	<*e*_1_,*d*_1_>; <*e*_2_,*d*_2_>	None
5	<*e*_2_,*d*_1_>; <*e*_1_,*d*_2_>	None
6	<*e*,*d*_1_>	<*loss,e,d*_2_>
7	<*e*,*d*_2_>	<*loss,e,d*_1_>
8	<*e*,*d*_1_>	None
9	<*e*,*d*_2_>	None
10	<*e*,*d*_1_>	None
11	*<e*,*d*_2_>	None
12	<*e*_1_,*d*>; <*e*_2_,*d*>	<*dupl,e,d*>
13	<*e*_1_,*d*>; <*e*_2_,*d*>	None
14	<*e*_1_,*d*>; <*e*_2_,*d*>	None
15	<*e*_1_,*d'*>; <*e*_2_,*d*>	<*tr*^*+*^*o,e*_1_*,d*>, <*tr*^*+*^*i,e*_1_*,d'*>
16	<*e*_2_,*d'*>; <*e*_1_,*d*>	<*tr*^*+*^*o,e*_2_*,d*>, <*tr*^*+*^*i,e*_2_*,d'*>
17	<*e*_1_,*d'*>; <*e*_2_,*d*>	<*gain,e*_1_*,d'*>
18	<*e*_2_,*d'*>; <*e*_1_,*d*>	<*gain,e*_2_*,d'*>
19	<*e*,d*>	<*sleep,e,d*>
20	*<e*_0_,*d'*>	<*gain_big,e,d'*>
21	*<e*,*d'*_1_>	<*tr*^*–*^*o,e,d*>, <*tr*^*–*^*i,e,d'*>, <*loss*^*–*^*,e,d*>
22	<*e*,*d'*_1_>	<*gain,e,d'*>
23	<*e*_1_,*d'*_1_>; <*e*_2_,*d'*_2_>	<*tr*^*–*^*o,e,d*>, <*tr*^*–*^*i,e,d'*>, <*loss*^*–*^*,e,d*>
24	<*e*_1_,*d'*_2_>; <*e*_2_,*d'*_1_>	<*tr*^*–*^*o,e,d*>, <*tr*^*–*^*i,e,d'*>, <*loss*^*–*^*,e,d*>
25	<*e*_1_,*d'*_1_>; <*e*_2_,*d'*_2_>	<*gain,e,d'*>
26	<*e*_1_,*d'*_2_>; <*e*_2_,*d'*_1_>	<*gain,e,d'*>
27	<*e*,*d'*_1_>	<*tr*^*–*^*o,e,d*>, <*tr*^*–*^*i,e,d'*>, <*loss*^*–*^*,e,d*>, <*loss,e,d'*_2_>
28	*<e*,*d'*_2_>	<*tr*^*–*^*o,e,d*>, <*tr*^*–*^*i,e,d'*>, <*loss*^*–*^*,e,d*>, <*loss,e,d'*_1_>
29	<*e*,*d'*_1_>	<*gain,e,d'*>, <*loss,e,d'*_2_>
30	<*e*,*d'*_2_>	<*gain,e,d'*>, <*loss,e,d'*_1_>
31	<*e*_1_,*d'*>; <*e*_2_,*d'*>	<*tr*^*–*^*o,e,d*>, <*tr*^*–*^*i,e,d'*>, <*loss*^*–*^*,e,d*>, <*dupl,e,d'*>
32	<*e*_1_,*d'*>; <*e*_2_,*d'*>	<*gain,e,d'*>, <*dupl,e,d'*>
33	<*e*_1_,*d'*>; <*e*_2_,*d"*>	<*tr*^*–*^*o,e,d*>, <*loss*^*–*^*,e,d*>, <*tr*^*–*^*i,e,d'*&*d"*>, <*tr*^*+*^*o,e*_1_&*e*_2_*,d'*&*d"*>, <*tr*^*+*^*i,e*_1_&*e*_2_*,d'*&*d"*>
34	<*e*_1_,*d'*>; <*e*_2_,*d"*>	<*gain,e, d'*&*d"*>, <*tr*^*+*^*o,e*_1_&*e*_2_*,d'*&*d"*>, <*tr*^*+*^*i,e*_1_&*e*_2_*,d'*&*d"*>

Consider *i* the ordering of events specified in Table 1; the second column specifies the termini of the edge projected from the pair <*e*, *d*>. The third column specifies triplets to be associated with edges of *R*_*j*_. A notation *e*_1_&*e*_2_ designates a multiplier of 0.5. In rows 1, 21, 23, 24, 27, 28, 31, 33 the triplets <*loss*^*–*^*,e,d*> designate the loss of the donor copy of the gene after transfer.

Denote a *T*-*type* a set *T* of edges with all descendant leaves marked with * in one or several trees *G*_*j*_ (a disjunctive union over *j*). An example is a set of ancestral ribosomal or mitochondrial genes.

Let *u* be a fixed tube. The given set *I* and tube *u* define the set *X* of edges in *R*_*j*_: edge *i* in DAG is included in *X* if one of the triplets at the intersection of the third column and the *i*-th row in Table 2 contains the first member belonging to *I* and the third member being the tube *u*. Denote this condition *i* ∈ *I,u*. Note that the second and third members of any triplet are uniquely determined by the terminus/termini of edge *i*, ref. to Table 2.

Analogously, given sets *I* and *T* define the set *X* of edges in *R*_*j*_: edge *i* in DAG is included in *X* if one of the triplets at the intersection of the third column and the *i*-th row in Table 2 contains the first member belonging to *I* and the second member being an edge from *T*. Denote this condition *i* ∈ *I, T*.

Compute expectations of the parameters of the stochastic process described in Second scenario design: a random process on the graph, the “amount of events from *I* in tube *u*” *f*(*I*, *u*) and the “amount of events from *I* on edges from *Т*” *g*(*I*, *Т*):

$$f\left(I,u\right)=\sum_{j}\sum_{e,d}\sum_{<e,d>\to i;\;i\in I,u}p\left(e,d,j,i\right)$$

where <*e*, *d* > → *i* signifies that edge *i* originates from vertex <*e*, *d* >. If *i* is one of the last two rows in Table 2, then in the notation *d'&d"* the summands for *u = d'* or *u = d"* are halved.

For the given *I* and *Т* the value of *g*(*I*, *Т*) is

$$g\left(I,T\right)=\sum_{j}\sum_{e,d}\sum_{<e,d>\to i;\;i\in I,T}p\left(e,d,j,i\right)$$

If *i* is one of the last two rows in Table 2, then in the notation *е*_1_*&е*_2_ the summands for *e*_1_ ∈ *T* or *e*_2_ ∈ *T* are halved.

In some cases, one may be interested to know the mathematical expectation of the total cost of events rather than their amount. The expectations are obtained using the formulas:

$$\begin{array}{c} \mathrm{cf}\left(I,u\right)=\sum_{j}\sum_{e,d}\sum_{<e,d>\to i;\;i\in I,u}c_{i}\cdot p\left(e,d,j,i\right)\\ \mathrm{cg}\left(I,T\right)=\sum_{j}\sum_{e,d}\sum_{<e,d>\to i;\;i\in I,T}c_{i}\cdot p\left(e,d,j,i\right) \end{array}$$

Under *k* =1 all expectations equal the number of events or the cost values.

More general characteristics can also be estimated, such as the sum

(6) $$\sum_{u,i\in I}\mathrm{cf}\left(I,u\right)$$

of expectations of the event costs over all tubes *u* and all events *i* from *I*, where *I* includes the gene gain (*gain*), origin of the common ancestor of all genes (*gain_big*), gene duplication (*dupl*), loss (*loss*), transfer from a tube (*tr*^*–*^*o* или *tr*^*+*^*o*), loss of the transferred copy in the donor (*loss*^*–*^). Other sets *I* can be used in (6).

Denote the sum (6) as the cost of the second scenario.

## Results and discussion

The models and algorithms described in the Methods are original developments of the authors and largely comprise the results of the study. This section details their implementation, testing on various data, and other relevant results.

### Implementation of the first algorithm

The Super3GL program accepts a set of rooted gene trees *G*_*j*_, which are allowed to contain polytomous vertices (ref. also to Additional file 1).

The program produces a supertree that amalgamates the set of input trees, allowing for duplications, gains, losses and horizontal transfers as evolutionary events, and imposes no condition on *P* (e.g. condition (*)); thus, the program realizes the heuristic algorithm described in Description of the first algorithm.

The input and resulting trees are in the Newick parenthesis format. If requested, the reliability of each supertree vertex is included in the tree notation as a length of the incoming edge; the general reliability of the supertree can also be computed.

Super3GL is written in C++ as a command-line utility and optionally accepts a configuration file to avoid re-typing non-default arguments. As mentioned above, the algorithm consists of two phases. Phase I, which *builds a set of basic trees*, cannot be interrupted. Phase II, which builds the *final supertree from the set of basic trees* incrementally by induction, is independent from the first phase and can be interrupted and resumed at any time.

The program automatically detects the MPI environment of version 1.2 or above; in which case it runs the parallel version of the algorithm. Detailed information about the program performance and scalability is given in the user’s manual.

Both 32-bit and 64-bit versions of Super3GL were tested on MS Windows and Linux on a stand-alone computer with 1–4 CPUs, as well as on the MVS-100K cluster of the Joint Supercomputer Center of the Russian Academy of Sciences [21] using up to 2048 CPUs.

The source code of Super3GL for Linux can be obtained free of charge from the Web page [19] under the GNU General Public License version 3.

### Implementation of the second algorithm

Embed3GL implements all operations discussed in The second algorithm: reconciliation of gene and species trees and building evolutionary scenarios. The program inputs a set of gene trees *G*_*j*_ that are allowed to contain polytomous vertices and paralogs. All trees are rooted, otherwise the algorithm from Additional file 1 is pre-applied.

The original species tree *S* and its modified version *S*_0_ are provided as one tree: the name of each vertex in the parenthesis notation of *S* is followed by an integer number, the “length” of the incoming tube. This value indicates the number of “new” tubes in *S*_0_ that form in the place of the “old” tube in *S* by inserting additional vertices. The default length of 1 means that no new vertex is inserted. A separate program, also available at the Web page [20], can be applied for time-slicing of a given species tree, which will be converted into the required tree format.

Each new tube *d* is attributed to a certain old tube, $\overset{Â¯}{d}=\overset{Â¯}{d}\left(d\right)$. It allows to compute characteristics of the old tube based on those of new tubes, which is frequently of interest. For instance, one may need $\sum_{d\in \overset{Â¯}{d}}f\left(d\right)$, where $d\in \overset{Â¯}{d}$ means that the new tube *d* is a part of the old tube $\overset{Â¯}{d}=\overset{Â¯}{d}\left(d\right)$, and *f* is the desired characteristic.

The Embed3GL program is written in C/C++ as a command-line utility and optionally accepts a configu-ration file to avoid re-typing of non-default arguments. The program automatically detects the MPI environment (version 1.2 or above), in which case it implements an effectively parallelized version of the algorithm.

The input gene trees are provided in the Newick parenthesis format as one or several files; the species tree is provided in the same notation in a separate file. All operations mentioned in The second algorithm: reconciliation of gene and species trees and building evolutionary scenarios can be performed serially or in any desired combination.

The Embed3GL program executables for 32/64-bit Windows along with the user’s manual and usage examples are freely available at the Web page [20]. The source code for Linux can be obtained free of charge from the same page under the GNU General Public License version 3.

### Testing of the algorithms

The Super3GL performance and results were compared against recognized supertree building programs on artificial and biological data. All comparisons were done in the uniprocessing mode on an Intel Xeon 2.0 GHz platform. Stochastic programs were run several times and the best result of the series was used for comparison. Super3GL was run once because its algorithm is deterministic. Selected comparisons with RFsupertrees [5] and Clann version 3.0.2 [22] are presented in Table 3. All programs were run with default parameter settings.

**Table 3:** Comparison of Super3GL with RFsupertrees and CLANN version 3.0.2

**Description**	**Super3GL**	**RFsupertrees**	**CLANN**
Artificial data (Additional file 3): 40 species, 1000 gene trees, 50932 leaves			
Supertree S*	Figure 7	Additional file 5	Additional file 6
Total cost of S*	97443	114028	158751
Cost of the second scenario	151630	173527	218958
Running time	21 m	10 m	847 m
Biological data (Additional file 4): 110 species, 3396 gene trees, 33470 leaves			
Supertree S*	Figure 8	Additional file 7	Additional file 8
Total cost of S*	210917	234880	234933
Cost of the second scenario	535524	660021	706826
Running time	14 m	107 m	2234 m

The total cost of the supertree and the cost of the second evolutionary scenario are defined with *c*({*G*_*i*_}, *S* *) and formula (6), respectively. Individual event costs are as follows: *c*(dupl) = 3, *c*(loss) = 2, *c*(gain) = 12, *c*(gain_big) = 10, *c*(sleep) = 20, *c*(tr_with) = 17.6, *c*(tr_without) = 19.6.

The three programs *used the same input files* provided in Additional file 3 (artificial data) and Additional file 4 (biological data).

#### Algorithms comparison with artificial data

**Figure 7:**
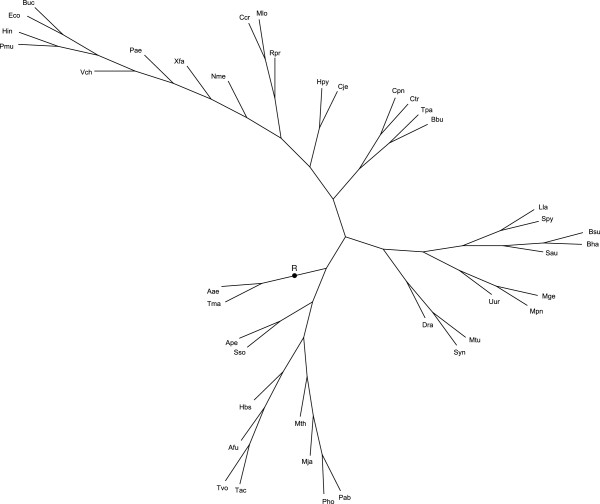
The artificial species tree S* used to simulate sets {*G*_***j***_**} of gene trees (40 species). **The tree root is denoted by *R*. One of the simulated sets {*G*_*j*_} is presented in Additional file 3. The Super3GL program applied to {*G*_*j*_} reconstructed the known supertree S* in 95% cases. The total mapping cost equals 97443. Leaf notations: Archaea: *Archaeoglobus fulgidus* (Afu), *Halobacterium sp. NRC-1* (Hbs), *Methanococcus jannaschii* (Mja), *Methanobacterium thermoautotrophicum* (Mth), *Thermoplasma acidophilum* (Tac), *Thermoplasma volcanium* (Tvo), *Pyrococcus horikoshii* (Pho), *Pyrococcus abyssi* (Pab), *Aeropyrum pernix* (Ape), *Sulfolobus solfataricus* (Sso); Gram-positive bacteria: *Streptococcus pyogenes* (Spy), *Bacillus subtilis* (Bsu), *Bacillus halodurans* (Bha), *Lactococcus lastis* (Lla), *Staphylococcus aureus* (Sau), *Ureaplasma urealyticum* (Uur), *Mycoplasma pneumoniae* (Mpn), *Mycoplasma genitalium* (Mge); α-Proteobacteria: *Mesorhizobium loti* (Mlo), *Caulobacter crescentus* (Ccr), *Rickettsia prowazekii* (Rpr); β-Proteobacteria: *Neisseria meningitidis MC*58 (Nme); γ-Proteobacteria: *Escherichia coli K12* (Eco), *Buchnera sp. APS* (Buc), *Pseudomonas aeruginosa* (Pae), *Vibrio cholerae* (Vch), *Haemophilus influenzae* (Hin), *Pasteurella multocida* (Pmu), *Xylella fastidiosa* (Xfa); ε-Proteobacteria: *Helicobacter pylori* (Hpy), *Campylobacter jejuni* (Cje); Chlamydia: *Chlamydia trachomatis* (Ctr), *Chlamydia pneumoniae* (Cpn); Spirohetes: *Treponema pallidum* (Tpa), *Borrelia burgdorferi* (Bbu); others: *Deinococcus radiodurans* (Dra), *Mycobacterium tuberculosis* (Mtu), *Synechocystis* (Syn), *Aquifex aeolicus* (Aae), *Thermotoga maritime* (Tma)*.*

*Artificial trees* were randomly generated from a known species tree S*. An example S* with 40 leaves is given in Figure 7. An example set {*G*_*j*_} of 1000 generated gene trees is given in Additional file 3. Trees contain 50,932 leaves in total. The method used to generate gene trees on a given species tree is described in [8], p. 166. As mentioned below, the procedure of trees simulation along a topology needs further study and justification.

Super3GL reconstructed the known species tree in 95% cases, *S* = S*. The two other programs used the same set of input trees but often constructed supertrees essentially different from S*; ref. e.g. to Additional files 5 and 6. The total costs of mapping of {*G*_*j*_} into *S* are as well presented in Table 3.

#### On the Robinson-Foulds distance

A natural approach to compare species trees constructed on the basis of an identical set of gene trees is to compare values of the total cost functional. Indeed, the supertree building problem is formulated (at least in this study) in terms of minimizing this functional. In essence, this functional is a measure of distance between the given set {*G*_*j*_} and the supertree *S*.

Different approaches to measure this distance are known. Thus, the *RF-functional*$\mathrm{RF}\left(\left\{G_{j}\right\},S\right)=\sum_{j}\mathrm{RF}\left(G_{j},S\right)$ is a sum of Robinson-Foulds distances [5,23] between *G*_*j*_ and *S* over all *G*_*j*_. A rigorous comparison between the functionals *RF*({*G*_*j*_}, *S*) over all *G*_*j*_. A rigorous comparison between the functionals *RF*({*G*_*j*_}, *S*) and $c\left(\left\{G_{j}\right\},S\right)=\sum_{j}c\left(G_{j},S\right)$ requires a separate systematic study. Below are some preliminary considerations.

Assume that tree *S* contains the set of leaves *V*_0_, and consider only species notations in leaves of *G*_*j*_. Typically, each *G*_*j*_ contains less species than *S*, and computing a *RF*-distance requires pruning of certain amount of species from *S* for each current *G*_*j*_. Properties of the *RF*-functional need to be studied.

Under the absence of paralogs, the minimization of the *RF*-functional is equivalent to maximization of clades matching between the topologies of *G*_*j*_ and *S*. In terms of mapping *α*, it is the maximization of cases when only one edge of the gene tree enters a tube of the species tree (i.e. the edge origin is mapped into the tube origin or earlier, and the edge terminus – into the tube or later). In biological terms, this speciation event is not associated with acquisition of paralogs. The authors are unaware of any research that interprets the *RF*-measure in terms of gene evolution events.

As with the mapping cost, the problem of minimizing the *RF*-functional is *NP*-hard, unless the tree *S* contains only clades belonging to a pre-defined set *P*. When this non-standard statement is assumed, the problem is solved with our algorithm exactly as described in this study for the cost functional. The proposed algorithm is universally applicable to any functional defined in terms of mapping edges. A natural example in case of paralogs is the minimization of the total amount of edges that enter tubes of the species tree. The described cost functional performs better than *RF-*functional even in the special case, where only gene duplications and losses are considered.

#### Algorithms comparison with biological data

*Biological data* is a set of unrooted gene trees provided by the courtesy of Prof. James McInerney (National University of Ireland, Maynooth). The trees were rooted using the procedure described in Additional file 1 to obtain the set of 3396 gene trees for 110 prokaryotic species. The trees contain 33,470 leaves in total. The set is provided in Additional file 4.

**Figure 8:**
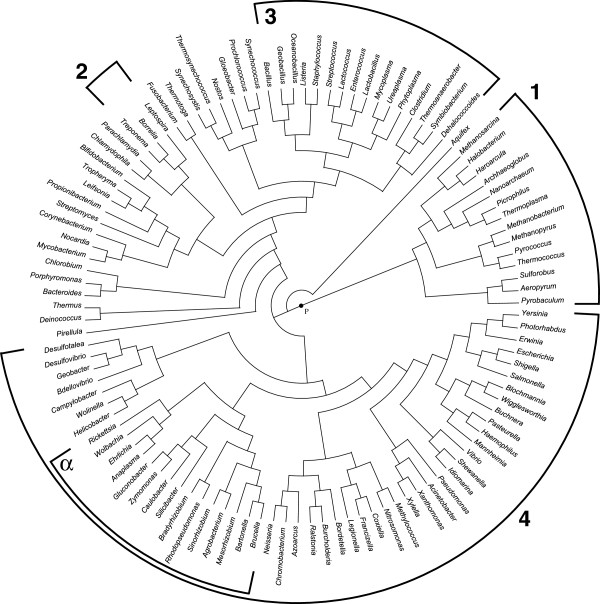
**The supertree built by Super3GL for biological data from Additional file **4**. **The tree root is denoted by *R*. Numbers indicate the phylogenetic patterns mentioned in Testing of the algorithms.

The supertree built by Super3GL is shown in Figure 8. It coincides mainly with the species tree from [24], with the same differences as between the tree of [24] and a later genomic tree of [25], which suggests support for our supertree building method. Supertrees built by the two other programs (ref. to Additional files 7 and 8) essentially differ from the mentioned trees [24,25].

Trees presented in Figure 8 and Additional files 7, 8 were not manually edited.

A comparative biological interpretation of our obtained supertree and the topology of other two trees also favors the Super3GL result. Consider four widely accepted phylogenetic patterns:

1) Archaebacteria and Eubacteria form two separate basal domains;

2) Spirochaetes are monophyletic within Eubacteria;

3) Bacilli, Clostridia, Lactobacilli, *Mycoplasma* and other Mollicutes constitute a separate monophyletic lineage within Eubacteria;

4) Proteobacteria are monophyletic within Eubacteria and contain the monophyletic subclade of *α*-Proteobacteria.

The tree in Figure 8 represents all four patterns. The tree in Additional files 7 contains only pattern 4, but splits Archaebacteria into a paraphyletic grade, separates spirochaetes (*Borrelia*, *Leptospira*, *Treponema*) among three distant lineages, places Clostridia+Mollicutes and Bacilli+Lactobacilli into different clades, the latter also containing a spirochaete *Treponema*. The tree in Additional file 8 does not show any of the four patterns: Archaebacteria are not basal, Spirochaetes largely intermix with other bacteria, Phytoplasma and Clostridia enter the Archaebacteria clade, Bacilli and Lactobacilli are mixed with Bacteroidetes, *Mycoplasma* – with selected Chlamydiae, most *α*-Proteobacteria are scattered between early diverging lineages, *Rickettsia* and *Ehrlichia* are placed in two different distant clades. All trees, however, show minor deviations from the biologically expected topology at a more shallow level. Thus, *Leifsonia* is always placed closer to *Bifidobacterium* than to other actinomycetes; in Figure 8 Pasteurellaceae enter Enterobacteriaceae. Such artifacts might indicate sampling errors of the data in Additional file 4.

Compare the evolutionary scenario designs defined in Methods. The two designs are compared in Table 4 on the basis of the same set of input gene trees.

**Table 4:** Example characteristics of the first and second scenario designs

	**Artificial data**		**Biological data**	
**Scenario characteristics**	**1st design**	**2nd design**	**1st design**	**2nd design**
Total cost / expectation	97443.4	151629.7	210917.0	535524.0
Total cost / expectation of gains	60.0	358.4	53448.0	77040.5
Total cost / expectation of losses	38024.0	56660.0	98376.0	187600.5
Total cost / expectation of duplications	26796.0	34324.6	38286.0	44639.6
Total cost / expectation of transfers	32563.4	60168.3	17887.0	223854.8
Total cost / expectation of the gain_big events	0.0	118.4	2920.0	2388.6
Running time	<1m	2m	15m	41m

Input tree data is the same as for Table 3. The tree *S* is obtained by the supertree building algorithm described in the paper. The degree of ramification *k* = 10. Individual event costs are as follows: *c*(dupl)=3, *c*(loss)=2, *c*(gain)=12, *c*(gain_big)=10, *c*(sleep)=20, *c*(tr_with)= 17.6, *c*(tr_without)=19.6. The running time is specified for parallel computations on a 16-CPUs platform. The cost in the second design and the expectation of the total event cost are defined in Table 3 and by formula (6), respectively.

Table 3 (the “cost of second scenario” row) details the comparison of the three programs. Note that comparing programs against the first and second scenarios produces the same result. Example expectations of the total (over all tubes) event costs for the two scenarios are given in Table 4.

Analyses used the NCBI taxonomy [26]. Trees were visualized with TreeView [27] and Dendroscope [28].

The rooting algorithm for unrooted trees is trivial and explained in Additional file 1.

## Conclusions

The problem of optimal amalgamation of a set of trees has a long history. This problem can be generalized into searching for an “average” graph of a given set of graphs. In the phylogenetic context, that will describe the desired supertree. Such graph will globally minimize the total sum of differences between each reconciled tree and the supertree. Pioneer studies (ref. to [2] and further references provided therein) defined the difference between the trees *G* and *S* in terms of the cost *с*(*G*, *S*) of mapping *α* of one tree into another. Under this concept, searching for a supertree was naturally viewed as searching for the global minimum of the functional $\underset{j}{\sum }c\left(G_{j},S\right)$ referred to as the *cost* of the amalgamation of trees *G*_*j*_.

The set of admissible trees *S* was not always explicitly specified for this functional. Its minimum was implied to be found among *all species trees* that contain species present in all amalgamated input trees. Under this statement, the problem cannot be rigorously solved in polynomial time.

We suggest a reformulation to search for the supertree among species trees that contain clades present in the set of input trees or, more generally, belonging to a predefined set *Р*. We developed a deterministic algorithm that finds the supertree for any given *P* in the time cubic of |*P*|. Moreover, for a special common case the algorithm was mathematically proved to find exactly the global minimum of the total amalgamation cost.

The software implementation of the developed algorithm performs faster and more accurately comparing to known tools of inferring supertrees. Empirical testing was done with artificial and biological data. However, for its rigorous statistical verification a sound comparative framework to cross-test supertree building algorithms is still to be developed.

Of basic importance to approach the tree amalgamation problem is to define evolutionary events that can biologically explain a correct amalgamation. The authors developed a detailed list of such events, which is far more extensive than found in current literature. The ultimate definition of an evolutionary scenario will require further research. We suggest two approaches to build scenarios. Their corresponding algorithms are mathematically proved and possess a cubic complexity to the input data size.

## Competing interests

The authors declare that they have no competing interests.

## Authors’ contributions

VAL and KYG proposed the model, definitions and statements, chose source data. VAL, KYG and LYR compared different tools. LIR wrote software and performed the computations. All authors wrote and approved the final manuscript.

## Authors’ information

VAL (alternative transcriptions of the last name: Lyubetskii, Liubetskii, Liubetskiii, Liubetskii) graduated from Moscow State University, Faculty of Mathematics and Mechanics, Ph.D. and D.Sc. in Math (theoretical computer science, mathematical logic, algebra and number theory), full professor.

LIR graduated from Moscow Institute of Electronics and Mathematics, Faculty of Applied Mathematics, Ph.D. in Tech (system analysis, information management and processing).

LYR graduated from Moscow State University, Faculty of Biology, Ph.D. in Life Sciences (molecular biology and evolution).

KYG graduated from Moscow State University, Faculty of Mathematics and Mechanics, Ph.D. in Math (mathematical logic, algebra and number theory).

The authors are affiliated with the Laboratory for mathematical methods and models in bioinformatics, Institute for Information Transmission Problems of the Russian Academy of Sciences (Kharkevich Institute), and with Moscow State University. Web: http://lab6.iitp.ru/en/

## Reviewers’ comments

Reviewer’s report 1

Prof. Anthony Almudevar

University of Rochester, United States of America

I have reviewed the paper and support publication, and have no specific comments.

Quality of written English: Acceptable

Reviewer’s report 2

Prof. Alexander Bolshoy (nominated by Prof. Peter Olofsson)

Institute of Evolution, University of Haifa, Israel

1) The authors propose a non-standard reformulation of the traditional NP-hard supertree building problem. Choosing a particular definition of the cost *c*(*G*, *S*) of mapping of a gene tree *G* into a species tree *S* the classical problem is to find such *S* that globally minimizes . I believe that Lyubetsky et al. propose natural reformulation of the classical problem. They propose to consider only such species trees *S* that contain clades present in input trees *G*_*i*_. However, it took me time to get to the conclusion that such reformulation is organic and follows from the evolutionary nature of the problem. I think that the authors should include a wordy informal explanation of the reformulation. This passage will help to non-mathematicians easier accept the contents.

Response. The described algorithm of supertree construction performs equally with any parameter *P*. Importantly, its runtime is cubic to the cardinality of *P*. If the set *P* contains all subsets of *V*_0_ our formulation coincides with the classical statement, and the supertree is not constrained in terms of its constituent clades. In this case, alike other algorithms, our algorithm becomes exponential to the size of input data. Its runtime can be set arbitrarily, in which case it will use a heuristic search and may not find the mathematically proved minimum of the functional.

The algorithm’s runtime becomes cubic if |*P*| is linear to the input data size, which is the case when *P* contains only clades present in all input trees. Biologically, this choice of *P* can be justified by constraining the supertree to contain only relationships present in the input data, thus not inventing artificial groupings of species. The correctness of the algorithm under this condition is the major hypothesis of the study. Its formal proof is not straightforward (at least to the authors), however it was empirically verified in this study on various data.

2) The authors have developed an algorithm to solve the supertree construction problem with time complexity *O*(*n*^3^). Description of the algorithm is long and difficult for understanding. It is OK but I would propose to add informal “popular-science” description in addition to the rigorous proof.

Response. *Intuitively, our algorithm of supertree construction resembles an algorithm of finding the minimum of a function of one variable defined on a segment. If solutions are known on two parts of the segment, the solution on their union can also be obtained. Analogously, if correct supertrees S*_1_* and S*_2_* defined on two disjoint subsets V*_1_ and *V*_2_*of the species set V*_0_*are known, the solution for the unionV*_1_∪*V*_2_* is also known, it is the joining of treesS*_1_ and *S*_2_* under the new root, Figure*3. If the set *V*_1_* is small (e.g., a triplet) then tree S*_1_*is found exhaustively. Remember that “treeS*_1_*is defined on set V*_1_” means that *V*_1_* is the set of species assigned to leaves ofS*_1_.* Such reduction fromV*_0_* toward subsets is not always possible as the subsets need to belong to a pre-defined setP at each step of reduction.*

*Parameter P is introduced to avoid the exponential growth of the variants space during the backward run of the algorithm from small subsets to total V*_0_, *which makes the algorithm’s runtime cubic to the size of |P|.*

*During phase I the algorithm constructs the master set of supertrees on subsets of the set V*_0_,* the basic trees on the basic subsets. During phase II (transition from T to S) the basic trees are used to compute the cost (or quality in Additional file*1) to choose the optimal extension of the current supertree *T*. The two alternative versions of phase II define this cost differently but both utilize the set of basic trees obtained during phase I.

3) A term “tube” appears on page 16 for the first time while the definition appears on page 20.

Response. Corrected. The term “tube” refers to an edge in the species tree to contrast the difference between edges in the species and gene trees. Edges of gene trees are visualized within the species tree tubes (Figures 5–6), which explains the etymology. Trees contain the root edge or the root tube (Figures 1–2).

4) On page 20 starts “the second algorithm”. I would propose to add informal “popular-science” description of the algorithm before introducing the terms “tube”, “scenario”, “evolutionary event”, etc.

Response. The algorithm of constructing supertrees is referred to as the “first algorithm”. “The second algorithm” is a collection of algorithms described under The second algorithm: reconciliation of gene and species trees and building evolutionary scenarios. It starts with the description of computing the originally introduced cost *c*(*GS*) of reconciling the gene and species trees. This algorithm is utilized in phase II of the first algorithm. The first algorithm can also be run with the classic cost *c*_0_(*GS*) defined [2], in which sense pre-applying “the second algorithm” is not mandatory. However, modeling shows that using the cost *c*(*GS*) produces more accurate results (data not shown).

Thus, numbering of the algorithms is conventional but their usage is mutual.

Each elementary evolutionary event described in Table 1 is supplied with a rule to compute the cost *c*(*e*,*d*,*i*) of the triplet <*e*,*d*,*i*>, where *i* is the initial event of the optimal (first) scenario that originates at the vertex <*e*,*d* >.

The idea behind computing the cost *c*(*G*,*S*) is similar to the one described in Response 2. If for *G*_1_ and *G*_2_ the costs *c*(*G*_1_,*S*), *c*(*G*_2_,*S*) are known and *G* is a disjunctive sum of sets *G*_1_, *G*_2_, i.e. *G*_1_∪*G*_2_, then the algorithm infers that the cost *c*(*G*_1_∪*G*_2_,*S*) equals *c*(*G*_1_,*S*)+*c*(*G*_2_,*S*)+*х*, where *х* is the total cost of elementary evolutionary events that occurred along the root edge *e* within tubes of *S* (Figure 5). Indeed, the costs of elementary events that occurred on edges of *G*_1_ and *G*_2_ already contributed to *c*(*G*_1_,*S*) and *c*(*G*_2_,*S*), respectively, and the costs of events that occurred on edge *e* are only accounted for by *x*. An example of events on edge *e* is given in Figure 5.

In its general design, the first scenario of the evolution of *G* into *S* is the mapping of edges of tree *G* inside the tubes of *S* such that the inferred distribution of elementary evolutionary events produces the minimal total cost.

The second scenario of the evolution of *G* into *S* is a probability-driven random walk process of edges inside the tubes, ref. to Response 5.

Thus, the “second algorithm” is a collection of algorithms of binarization, computing the cost *c*(*G*,*S*), computing the first scenario (the event tree *T*), computing the second scenario (random process on graph *R*) and its stochastic characteristics.

5) I’m afraid that subsection 4.4 is too important to be so short. However, it is possible that a “popular-science” addition mentioned above would easier reading of this subsection.

Response. *Second scenario design: a random process on the graph* contains a description of the stochastic process that defines the second evolutionary scenario. The process initiates at the root edge *e*_0_ inside the root tube *d*_0_ (i.e., in state <*e*_0_, *d*_0_>). Assume that edge *e* is located inside tube *d*, and the state <*e*,*d*> is not a *terminal pair*, i.e., *e* and *d* are not leaves cohered by the gene-species correspondence. Then, an event *i* that occurs on edge *e* inside tube *d* is selected from Table 1 based on the distribution of probabilities *p*_*i*_ over all triples <*e*,*d*,*i*>. The probabilities are pre-calculated and are the higher the lower are the costs *c*(*e*,*d*,*i*) of the first scenarios initiated with the event *i* in the state <*e*,*d*>. If the selected event *i* does not contain a bifurcation (ref. to Table 1) then the process proceeds from <*e*,*d*> into the state specified in the event. If the event *i* contains a bifurcation, then the process bifurcates into the two states specified in the event. The branching process ends by reaching all terminal pairs. Mathematical expectations of the amounts and costs of various event types in tubes in this stochastic process are estimated in Stochastic characteristics of the second scenario design.

Quality of written English: Acceptable

Reviewer’s report 3

Prof. Marek Kimmel

Rice University, United States of America

1) This is an interesting paper on an important subject, containing potentially important results. However, the style in which it is written makes understanding its message very difficult. Definitions are mixed with results and discussions and part of the results and arguments are concealed in non-mainstream publications. I encourage the authors to reorganize the paper thoroughly.

Response. The authors made all efforts to restructure the text to make it more clear, and added three illustrations. This study indeed operates with many concepts and definitions that, we hope, are clarified in responses to the reviews. The “Background and Problems” section introduces novel definitions and hypotheses not described in previous publications of the authors, and is followed by the “Methods” and “Results”. Description of the first algorithm and 1,3 of the Results contain the description and thorough testing of the first algorithm (constructing supertrees); reference [7] describes the class of algorithms, for which we prove the theorem mentioned in Response 7; the details and computer realization of one such algorithm provided in the paper are novel. The second algorithm: reconciliation of gene and species trees and building evolutionary scenarios introduces the second algorithm and detailed rationale behind it (ref. to Response 4 to the Reviewer 2). In reference [3] this algorithm was discussed in its perspective to solve a narrower scope of biological problems, with no mathematical details or a computer implementation. The mutual usage of the two algorithms is explained in Response 4 to Reviewer 2.

Some specific points are listed below.

2) “we believe that NP ≠ P”; please clarify (I am not sure of this is a question of beliefs).

Response. Corrected. We believe that the statement NP ≠ P is true. Even if NP = P, known algorithms do not solve the supertree finding problem in polynomial (particularly cubic) time.

3) The definition of clade (and other definitions) is placed after clades are mentioned.

Response. The definition of clade is introduced in the Background at its first appearance. In the Abstract this term is used in the common biological sense as a set of leaves-species descending from a tree vertex. The Abstract contains several terms that are all defined in the main text.

4) “With the standard event set and condition (*), the algorithm was mathematically proved [7]”; this statement leaves the reader in the dark concerning the exact statement of the algorithm that has been demonstrated. It should be stated clearly, as a mathematical theorem, with an explicit list of hypotheses and assertions.

Response. Reference [7] contains a 15-pages proof of the theorem that asserts for a class of algorithms (including the discussed algorithm of supertree construction):

if only duplications and losses are the allowed evolutionary events, and condition (*) is true, then any algorithm of this class exactly finds the global constrained minimum of functional (1). The constraint imposes the condition that all clades in the supertree belong to a pre-defined set *P*.

5) In particular, the status of the present paper compared to material in references [3] and [7] (which probably are not easy to obtain), is undefined. If these references can be treated as preliminary publications, I am inclined to advise that the an extract of the argument proving the algorithm be reiterated in the present manuscript. As it is now, the manuscript is a mosaic of references and it is not easy make sense of what is original and what is not. Basic background definitions should be listed in one section and not provided “on the go”.

Response. In the strict sense, absolutely original in this study is the description of the two programs [19,20] and their testing. The second program is an integral part of the first program (ref. to Response 4 to Reviewer 2), and therefore they are tested in complex.

With full respect, we believe that accumulating all definitions in one place will make the text less comprehensive: as for now, each of them appears exactly before it is used in the relevant context.

6) “It is difficult however to mathematically prove the algorithm for the case of the extended event set and/or a relaxation of condition (*). We believe that including horizontal gene transfers still produces valid results [7], and condition (*) can be relaxed.”; how does this relate to the exact version of the algorithm which is contained in the manuscript? Is the case considered here the one for which a mathematical proof is missing?

Response. For this algorithm it is yet unknown if the theorem discussed in Response 4 is true under the relaxation of its condition (e.g., allowing certain horizontal transfers). Therefore this algorithm remains heuristic, as stated in the text.

7) Subsequent paragraphs are arranged in an order which does not help understanding. For example, Algorithm 2 should be defined in a Methods section at the beginning of the paper. Customary subdivision into Introduction, Background, Methods and Data, Results and Discussion will in my opinion help the reader to understand what the paper is about.

Response. With full respect, the authors ask to retain the order, in which the algorithms are introduced. This point is justified in Response 4 to Reviewer 2.

Quality of written English: Not suitable for publication unless extensively edited.

Response. The text was reviewed by a native speaker.

## Supplementary Material

Additional file 1 **Rooting algorithm for unrooted trees. **Computational complexity of the first algorithm and reliability of the supertree. Alternative design of Phase II. Click here for file

Additional file 2 **Transition from a polytomous to binary tree. **Inductive step of constructing a directed acyclic graph. Click here for file

Additional file 3 **Input gene trees (artificial data). **(viewable by e.g. TreeViewX). Click here for file

Additional file 4 **Input gene trees (biological data). **(viewable by e.g. TreeViewX). Click here for file

Additional file 5 **Supertree built by RFsupertrees for artificial data from Additional file 3. **In the unrooted topology, the two outlined subtrees swapped with respect to the correct tree in Figure 7. The total mapping cost is 114028. Click here for file

Additional file 6 **Supertree built by CLANN version 3.0.2 for artificial data from Additional file 3. **In the unrooted topology, the two set-off edges are misplaced with respect to the correct tree in Figure 7. The total mapping cost is 158751. Click here for file

Additional file 7 **Supertree built by RFsupertrees for biological data from Additional file 4. **The tree root is denoted by *R*. Click here for file

Additional file 8 **Supertree built by CLANN version 3.0.2 for biological data from Additional file 4. **The tree root is denoted by *R*. Click here for file
