# Supplementary material for: Cubic time algorithms of amalgamating gene trees and building evolutionary scenarios
Source: Biol Direct. 2012 Dec 22;7:48. doi: 10.1186/1745-6150-7-48 (PMC3577452; doi:10.1186/1745-6150-7-48)
Supplement: Additional file 1 — Rooting algorithm for unrooted trees. Computational complexity of the first algorithm and reliability of the supertree. Alternative design of Phase II. [file 1745-6150-7-48-S1.doc]

# Supplement 1 – Rooting algorithm for unrooted trees. Computational complexity of the first algorithm and reliability of the supertree. Alternative design of Phase II

**Rooting algorithm for unrooted trees**. This algorithm is trivial but its implementation can be useful[[1]](#footnote-2). Let *G* be an unrooted tree, and each leaf of *G* be assigned a species name. For simplicity it is assumed that all species belong to one of the two non-overlapping “ancient” taxa, and each leaf is also assigned a name of its containing taxon. The aim is to insert the root on the edge that splits these taxa. In order to find this edge, the following characteristic *d* is computed for each edge in *G*.

Let *e* be an edge connecting vertices *u* and *v*. This edge splits *G* in two parts, the one denoted *A* that adjoins *u*, and the other denoted *B* that adjoins *v*. We denote by *b_ui_* and *b_vi_* the numbers of leaves from the *i*-th ($i=\overline{1,2}$) taxon in parts *A* and *B*, respectively. Let *I_u_* and *I_v_* be the overall numbers of leaves in parts *A* and *B*, respectively. Then *d_ui_* = *b_ui_* /*I_u_* is a share of leaves from the *i*-th taxon in part *A*, and *d_vi_* = *b_vi_* / *I_v_* — the same in part *B*. The edge characteristic is calculated as

$d(e\text{)}=\min\left\{\left(\sqrt{d_{u1}}-\sqrt{d_{v1}}\right)^{2},\left(\sqrt{d_{u2}}-\sqrt{d_{v2}}\right)^{2}\right\}$.

The sought-for edge *e*_0_ is such, at which the value *d*(*e*_0_) = *d*_max_ reaches its maximum. Let *d*_prmax_ be the value of *d*(*e*) closest to *d*_max_ (if the same *d*_max_ is reached for several edges, *d*_prmax_ = *d*_max_). Define the characteristic

$p(G)=\sqrt{d_{\max}}-\sqrt{d_{\text{pr}\max}}+\left(d_{\max}\right)^{2}$.

If *p*(*G*) exceeds the threshold *p* (the algorithm parameter), the root of *G* is placed on the edge *e*_0_. Otherwise, the algorithm reports no possible rooting. The value *p*(*G*) is called the *rooting quality* of tree *G*.

In some cases, leaves of the tree become assigned to more than two “ancient” taxa. If so, these taxa are sorted such that distant taxa appear in different groups, and the above described procedure is re-applied.

It often makes sense to prune low-supported edges off tree *G* before rooting. If pruning is requested, one unrooted tree can result in several rooted trees.

This algorithm was implemented as a collection of php scripts freely available at the Web page [19].

**Complexity**. The runtime of Phase II of the algorithm under the standard *P* has the order of *O*(*n·m*^4^) (if $\beta$ is calculated as in [2], henceforth referred to as “case A”) or the order of *O*(*n*·*m*^6^) (if $\beta$ is calculated as in [3], henceforth referred to as “case B”), and under an arbitrary *P* it has the order of *O*(|*P*|·*m*^3^) (case A) or the order of *O*(|*P*|·*m*^5^) (case B). In case of the standard *P* the corresponding runtime *n*·*m*^6^ of Phase II does not exceed the runtime *m*^3^*n*^3^ of Phase I if the number of trees *n* is not less than *m*^3/2^, [8]. The latter can be true for example, for 100 species and 1000 gene trees.

The total runtime of both phases under the standard *P* has the order of *O*(*n*·*m*^3^(*n*^2^+*m*)) (case A) or *O*(*n*·*m*^3^(*n*^2^+*m*^3^)) (case B), and under any *P* it has the order of *O*(|*P*|^3^+|*P*|^2^*nm*+|*P*|·*m*^3^) (case A) or *O*(|*P*|^3^+|*P*|^2^*nm*+|*P*|·*m*^5^) (case B).

In case of large input data Phase II can be realized with a simpler algorithm. The idea behind it is to compute the quality score of tree *S* not based on mapping but on its underlying topologies.

A topology {*a*, *b*, *c*} is equivalent to a tree with three leaves, *a*, *b*, and *c*. Define the *quality* *Q*(*t*) of topology *t* = *t*(*a,b,c*) for a triplet of leaves (i.e., species) *a, b, c* with the formula

$Q(t)=\frac{\sum_{k\in K} w(V_{k})}{\sum_{m\in M} w(V_{m})+\sum_{n\in N} w(V_{n})+1}$,

where *K* is a set of basic trees that contain the triplet and induce the topology *t* in it; *M, N* are sets of basic trees that also contain the triplet but induce two alternative topologies in it. Given a species tree *S*, the total quality of topologies *t* = *t*(*a,b,c*) induced by *S* for all triplets {*a*, *b*, *c*} of leaves-species, *a, b, c* in *S* is called the *quality Q*(*S*) of the tree *S*, i.e.,

$Q(S)=\sum_{\{a,b,c\}\in S} Q\left[t(a,b,c)\right]$.

**Reliability.** The *vertex reliability* in the final supertree *S* and the overall *reliability* of *S* are estimated as follows. Let *R* be a clade corresponding to a given inner vertex *s*. Denote *R*(*V*_0_) a set of all triplets {*a*,*b*,*c*} of species from *V*_0_ that have only two member species belonging to *R*. For a triplet {*a*,*b*,*c*} from *R*(*V*_0_) denote *R*{*a*,*b*,*c*} a unique topology on {*a*,*b*,*c*} such that the two elements of {*a*,*b*,*c*} belonging to *R* are siblings in *R*{*a*,*b*,*c*}. The ratio

$v(s)=\frac{\sum_{\{a,b,c\}\in R(V_{0})} Q\left(R\{a,b,c\}\right)}{\sum_{\{a,b,c\}\in R(V_{0})} \sum_{t} Q(t)}$,

where *t* runs over all topologies on {*a*, *b*, *c*} is called the reliability of vertex *s*. If the denominator of this ratio equals zero for vertex *s* (in which case the numerator is zero too), it is assumed that *v*(*s*) = –1. The minimum reliability over all vertices in *S* gives the reliability of *S*.

**Alternative design of Phase II**. Assume that all trees are orientated downwards from the root, and let *V*_0_ be a set of all species from all *G_i_* as above. A three-leaved seed tree induces a topology *t*(*a,b,c*) of maximum quality over all topologies of all triplets of species from *V*_0_. Each edge in the current partial supertree *S* is attempted for insertion of a new vertex *a* connected to each of the remaining species *s* from *V*_0_, and also for placing of a new root *a* above the current root, Fig. 4. Among such possible extensions *T* of *S*, we choose the tree *T* of the maximum quality $Q(T)$; it supersedes the current partial supertree *S*. Extensions are attempted until all species from *V*_0_ are added in the current tree, and the algorithm halts. The end of alternative Phase II.

Further research is needed to identify cases when the two alternative designs of Phase II produce different results.

1. All notations, references and citations in this Supplement are as in the main paper. [↑](#footnote-ref-2)
