# Supplementary material for: Cubic time algorithms of amalgamating gene trees and building evolutionary scenarios
Source: Biol Direct. 2012 Dec 22;7:48. doi: 10.1186/1745-6150-7-48 (PMC3577452; doi:10.1186/1745-6150-7-48)
Supplement: Additional file 2 — Transition from a polytomous to binary tree. Inductive step of constructing a directed acyclic graph. [file 1745-6150-7-48-S2.doc]

# Supplement 2 – Transition from a polytomous to binary tree. Inductive step of constructing a directed acyclic graph

**Transition from a polytomous to binary tree (the binarization operation)**.

Let *G* be a polytomous tree. We like to get the binary tree *G'* that is “equivalent” to *G*. If the procedure described in[[1]](#footnote-2) Section 2.1 encounters a polytomous edge *e*, denote *Е** a set of its descendant edges. Let *E* be an arbitrary non-empty subset in *E**. All such *E* are visited in the order of ascending cardinalities |*E*| (in the arbitrary order if cardinalities are equal), and in each *E* all tubes *d* are visited in the order defined in Section 2.1.

For singleton sets *Е*={*e*_1_} the cost *с*_min_(*E*,*d*) equals min*_i_* *с*(*e*_1_,*d*,*i*), where *i* runs over all rows in Table 1 (the start of induction).

For non-singleton sets *Е* the cost *с*_min_(*E*, *d*) is obtained as follows. All possible partitions of *E* into two non-empty subsets *E*_1_ and *E*_2_ are tried. Let *с*_min_(*E*,*d*) equal

$c_{\min}(E,d)=\min_{E_{1}+E_{2}=E} \min_{i} c(E,d,i)$, (**)

where *i* runs over all rows in Table 1, and *с*(*E*,*d*,*i*) is computed according to the corresponding formula (last column of Table 1) with *с*_min_(*E*_1_,*·*) and *с*_min_(*E*_2_,*·*) already known (the dot stands for an arbitrary tube).

The minimum of (**) is attained at a certain triplet <*E*_1_*'*,*E*_2_*'*,*i'*>. Denote the pair <*E*_1_*'*,*E*_2_*'*> a *minimal partition*, the row number (i.e. the event) *i'* – a *minimal row*, the parameter (a tube or a pair of tubes) at which the minimum is attained – a *minimal parameter*. The first and third columns of Table 1 contain the names of the event (the algorithm mainly uses the first column), the second column of Table 2 determines the *minimal parameter* for each row.

A pair <*E*,*d*> is assigned the cost *с*_min_(*E*, *d*), the event name and the minimal parameter. If the minimum is attained at several Table rows, the upper row is selected; if several partitions of equal cost correspond to the minimal row, one is selected arbitrarily.

The below assumption exists in formula (**). Some event types do not involve a bifurcation of *e* into *e*_1_ and *e*_2_ but are still tried in computing min*_i_* over *i*. If the minimal row corresponds to one such event, the corresponding pair <*e*,*d'*> determines the minimal parameter and is denoted by <*E*,*d'*>. There is no partitioning in this case.

An analogous procedure is applied to any binary edge *e*.

Define

*с*_min_(*e*,*d*) = *с*_min_(*E**,*d*).

The last *с*_min_(*e*_0_,*d*_0_) is computed by induction and called the *cost* of a *polytomous* gene tree *G* against the tree *S* and is denoted *с*(*G*, *S*).

It is easy to prove that this cost is a global minimum among all costs of possible binarizations of all polytomous vertices in *G*, and the minimum is attained exactly at the constructed binarization *G'*. It is easily proved that the costs of *G* of and *G'* coincide.

The backward run of the algorithm starts from the pair <*e*_0_,*d*_0_> and also visits edges newly added in *G* during the forward run. For the pair <*е*,*d*> with a polytomous *e*, in the case of partitioning the *new* (descending) edges are denoted *е*_1_ =*E*_1_*'* and *е*_2_ =*E*_2_*'*, and assigned pairs <*е*_1_,*d*_1_*'*> and <*е*_2_,*d*_2_*'*>, respectively. Otherwise, under no partition the endpoint vertex of *e* is assigned the pair <*e*,*d'*> and no new edges. The cases are selected depending on whether the event type chosen for pair <*е*,*d*> implies a bifurcation. This describes one step of the binarization of *G* into *G*'.

When the binarization is done, edges with certain *e* constitute a path, which is merged into a single edge, and intermediate edge information is removed.

**Induction step in the construction of a directed acyclic graph** (DAG).

The third column of Table 2 specifies triplets of objects: a tag, an edge in *G*, a tube in *S*_0_. Termini of edges projected from < *e*, *d*> during induction are specified in the second column of Table 2.

For each pair <*е*,*d*> visited as described in Section 2.1 find *k* events *i* (i.e., rows in Table 1) with minimal costs computed according to their parameters. If the total number *l* of events < *k*, all events are considered. For each row, one or two pairs are specified in the second column of Table 2. In the case of one pair, a unary (regular) edge is projected into this pair from <*е*,*d*> and is assigned the name of the event from the first column of Table 1. If two pairs are specified, a binary edge is projected into these pairs from <*е*,*d*>, and both constituent edges are assigned the name of the event from the first column. If the pair/pairs contain *d'* or *d'* and *d''*, then the projected edge depends on tube *d'* or tubes *d'* and *d''*. At this point DAG is constructed without numbers assigned to its edges.

The costs *c*(*e*,*d*,*i*) of selected events *i* are used to estimate values *p_i_* with the formula (for each DAG vertex where *i* enumerates outbound edges):

$\sum_{i=1}^{k} p_{i}=1$, $\frac{p_{i}}{p_{s}}=\frac{(c(e,d,s))^{3}}{(c(e,d,i))^{3}}$,

and to assign value *p_i_* to the *i*-th edge.

If for an *i*-th edge *c*(*e*,*d*,*i*) is 0, assume *p_i_* = 1, and *p_s_* = 0 for all other *s*. Only such edge is kept and assigned the value of 1. After completing the induction, the DAG construction is finished. The end of the **forward run** of the algorithm.

During the **backward run**, the algorithm visits vertices in the reverse order of visiting pairs <*е*,*d*>; *i*-s are tried as in the forward run.

Each vertex <*e*,*d*> and its outbound edge *i* are assigned values by induction. They are denoted *p*(*e*,*d*) and *p*(*e*,*d*,*i*), respectively, where *j* is the number of tree *G*.

For the root vertex define *p*(*e*_0_,*d*_0_) = 1, and for its outgoing edges *p*(*e*_0_,*d*_0_,*i*) equal *p_i_*.

For vertex <*e*,*d*> define $p(e,d)=\sum_{r\to<e,d>} p(r)$, where *r* runs over all ingoing edges; a binary edge is considered ingoing if either of its constituent edges enters the vertex. Each outbound edge *i* from <*e*,*d*> is assigned value $p(e,d,i)=p(e,d)\cdot p_{i}$. The end of the backward run and the algorithm of DAG construction.

1. All notations, references and citations in this Supplement are as in the main paper. [↑](#footnote-ref-2)
