# Supplementary figures and images for: Cubic time algorithms of amalgamating gene trees and building evolutionary scenarios
Source: Biol Direct. 2012 Dec 22;7:48. doi: 10.1186/1745-6150-7-48 (PMC3577452; doi:10.1186/1745-6150-7-48)

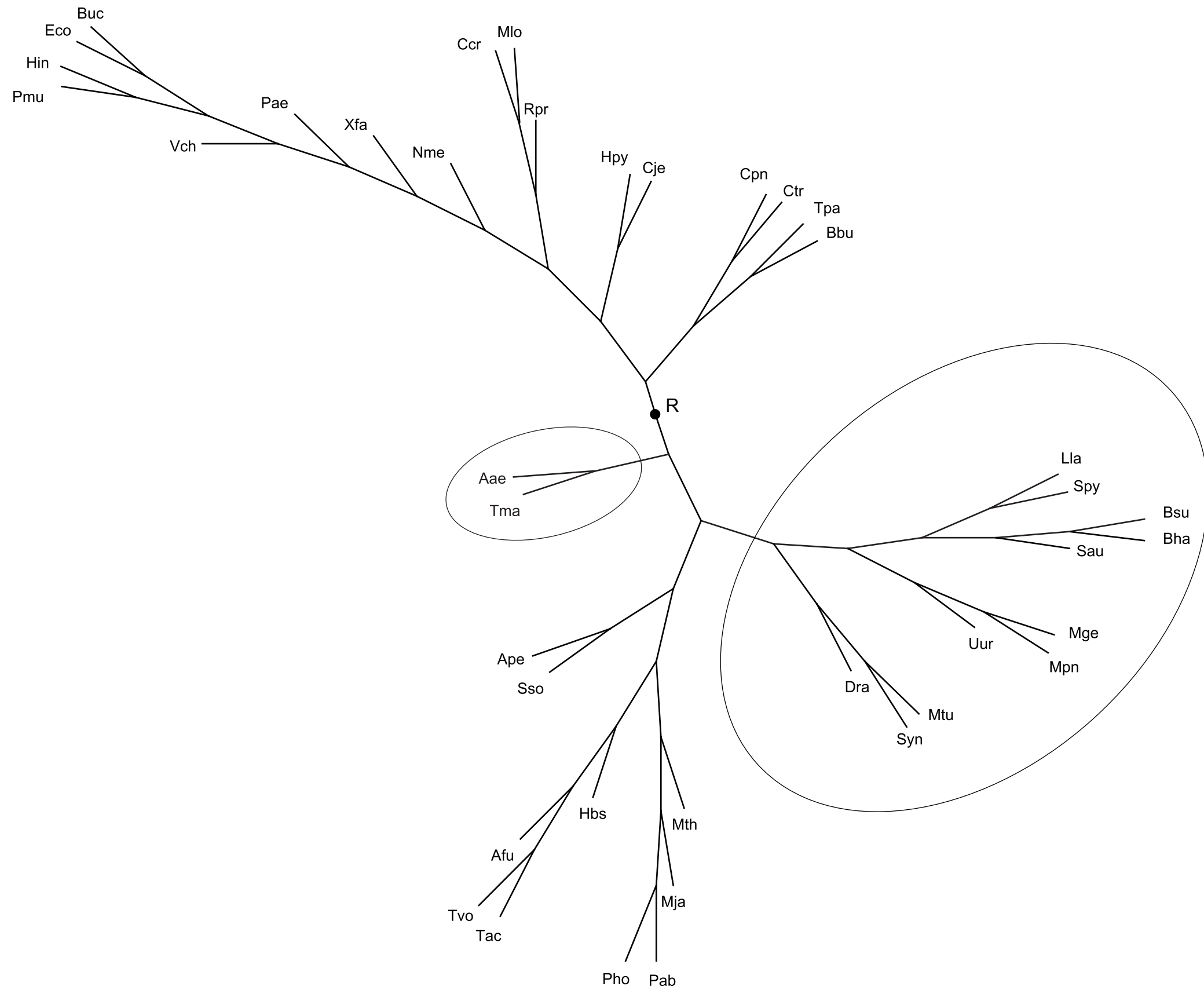

Supplement: Additional file 5 — Supertree built by RFsupertrees for artificial data from Additional file 3. In the unrooted topology, the two outlined subtrees swapped with respect to the correct tree in Figure 7. The total mapping cost is 114028. [file 1745-6150-7-48-S5.pdf]

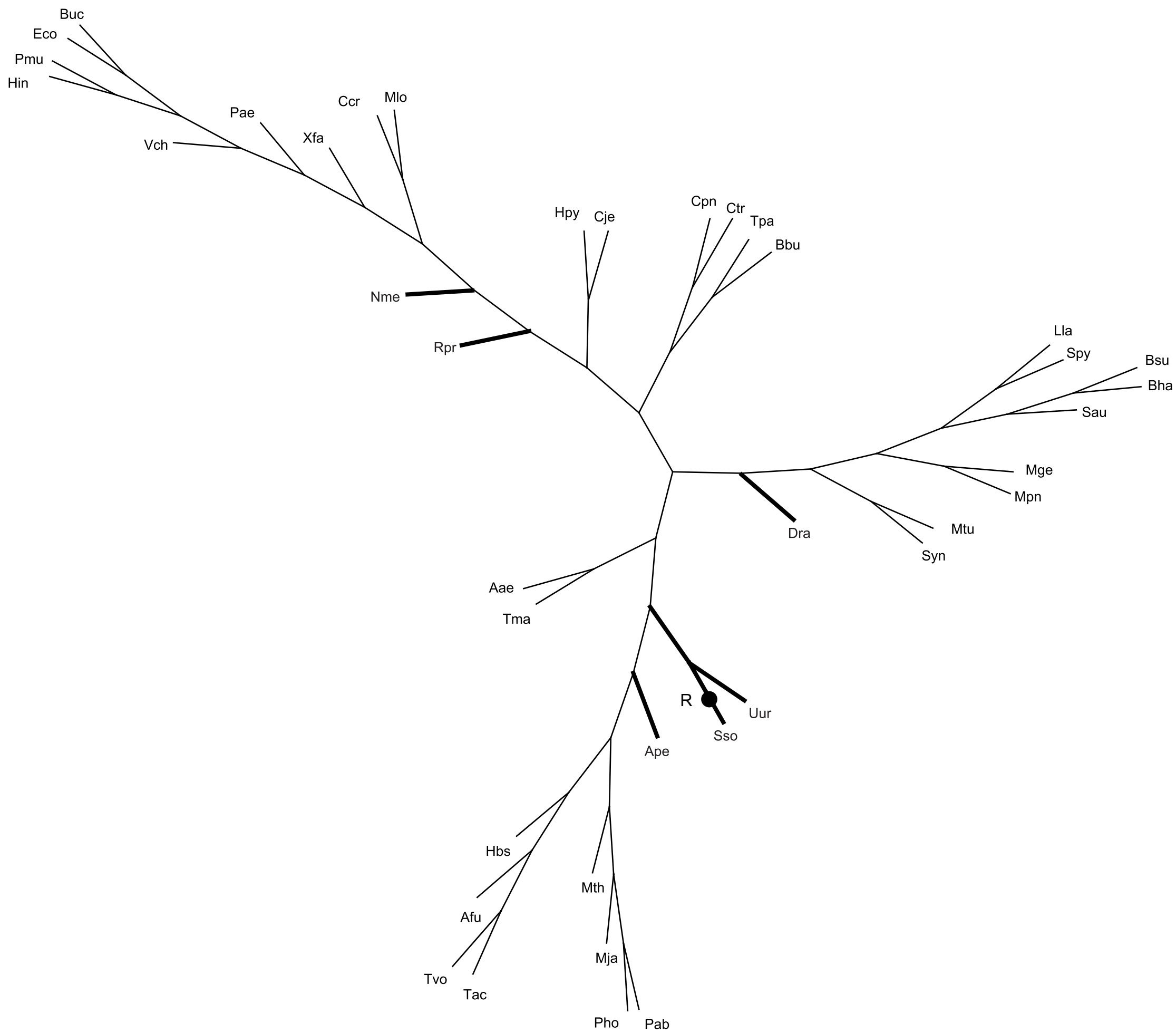

Supplement: Additional file 6 — Supertree built by CLANN version 3.0.2 for artificial data from Additional file 3. In the unrooted topology, the two set-off edges are misplaced with respect to the correct tree in Figure 7. The total mapping cost is 158751. [file 1745-6150-7-48-S6.pdf]

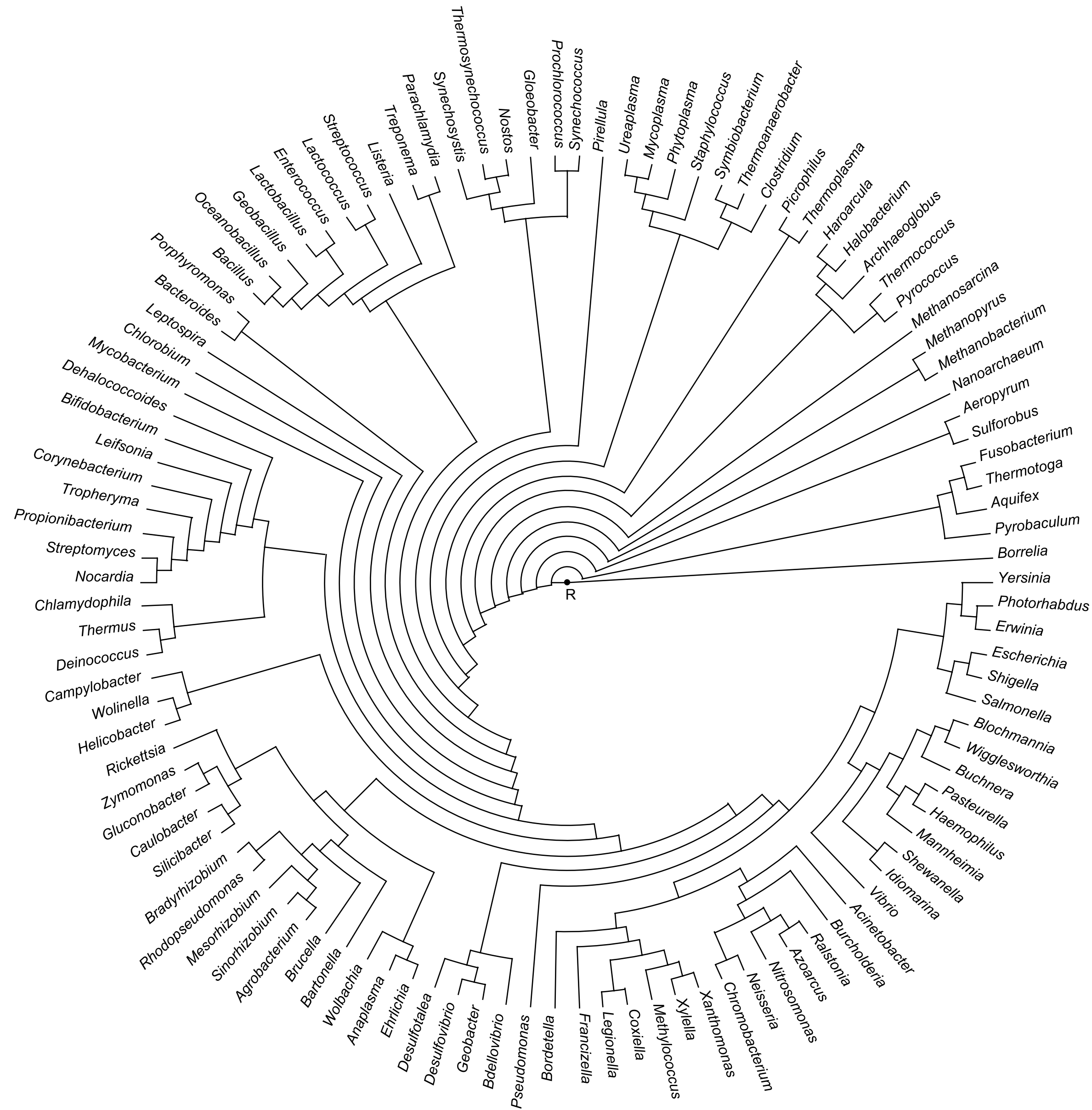

Supplement: Additional file 7 — Supertree built by RFsupertrees for biological data from Additional file 4. The tree root is denoted by R. [file 1745-6150-7-48-S7.pdf]

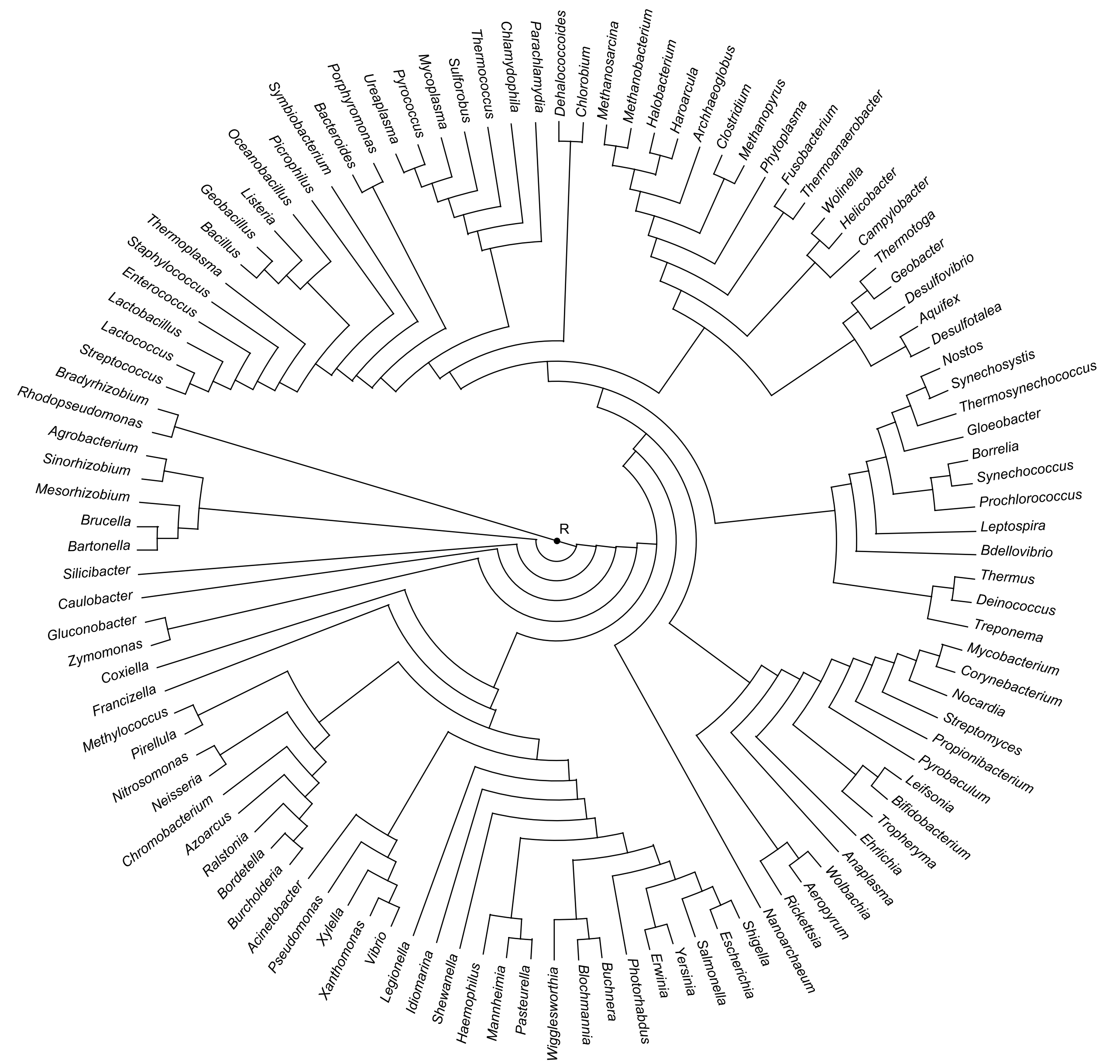

Supplement: Additional file 8 — Supertree built by CLANN version 3.0.2 for biological data from Additional file 4. The tree root is denoted by R. [file 1745-6150-7-48-S8.pdf]
